# Genome-wide comparison reveals large structural variants in cassava landraces

**DOI:** 10.1186/s12864-025-11523-y

**Published:** 2025-04-10

**Authors:** Michael Landi, Anna Vittoria Carluccio, Trushar Shah, Adnan Niazi, Livia Stavolone, Laurent Falquet, Andreas Gisel, Erik Bongcam-Rudloff

**Affiliations:** 1 Department of Animal Biosciences, Bioinformatics Section, Swedish University of Agricultural Sciences, Uppsala, Sweden; 2 International Institute of Tropical Agriculture, Nairobi, Kenya; 3 International Institute of Tropical Agriculture, Ibadan, Nigeria; 4 Institute of Sustainable Plant Protection, CNR, Bari, Italy; 5 Department of Biology, University of Fribourg, Fribourg, Switzerland; 6 Swiss Institute of Bioinformatics, Lausanne, Switzerland; 7 Institute of Biomedical Technologies, CNR, Bari, Italy

**Keywords:** Cassava, Structural variants, Chromosome 12, Large highly repetitive insert

## Abstract

**Background:**

Structural variants (SVs) are critical for plant genomic diversity and phenotypic variation. This study investigates a large, 9.7 Mbp highly repetitive segment on chromosome 12 of TMEB117, a region not previously characterized in cassava (*Manihot esculenta* Crantz). We aim to explore its presence and variability across multiple cassava landraces, providing insights into its genomic significance and potential implications.

**Results:**

We validated the presence of the 9.7 Mbp segment in the TMEB117 genome, distinguishing it from other published cassava genome assemblies. By mapping short-read sequencing data from 16 cassava landraces to TMEB117 chromosome 12, we observed variability in read mapping, suggesting that while all genotypes contain the insertion region, some exhibit missing segments or sequence differences. Further analysis revealed two unique genes associated with deacetylase activity, HDA14 and SRT2, within the insertion. Additionally, the *MUDR-Mutator* transposable element was significantly overrepresented in this region.

**Conclusions:**

This study uncovers a large structural variant in the TMEB117 cassava genome, highlighting its variability among different genotypes. The enrichment of HDA14 and SRT2 genes and the *MUDR-Mutator* elements within the insertion suggests potential functional significance, though further research is needed to explore this. These findings provide important insights into the role of structural variations in shaping cassava genomic diversity.

**Supplementary Information:**

The online version contains supplementary material available at 10.1186/s12864-025-11523-y.

## Background

Exploring comparative genomics across plant species holds great significance, offering valuable insights into genetic diversity, gene functionalities, and evolutionary studies. This exploration proves relevant in detecting various genomic variations with profound implications for the plants’ phenotypic traits. The understandings gained in these investigations are essential for improving crops and creating varieties with enhanced nutritional content, resilience against diseases, and adaptability to diverse environmental conditions.

DNA structural variants (SVs) exceeding 50 bps in plant genomes are still being explored despite their crucial influence on genomic diversity. These diverse SV types include insertions, deletions, translocations, and inversions [1]. Such structural variations can significantly impact the structure and function of genomes. Various mechanisms can contribute to forming SVs [2]. One mechanism involves TEs generated as insertion/deletion polymorphisms spanning several kilobase pairs characterized by their repetitive sequences, which can mediate ectopic recombination events, leading to even larger SVs [3]. Single nucleotide polymorphisms (SNPs) capture specific genomic variations. In contrast, SVs account for a larger proportion of heritable genetic variation and significantly impact the genome’s structure and function [4, 5]. SVs have become valuable in plant breeding [6, 7]. Within plants, SVs have been linked to variations in critical phenotypic traits like fruit color [1], fruit shape [8], and leaf size [9]. In studies involving tomatoes [6] and grapes [1], substantial impacts on important agricultural traits may arise from large genomic SVs. One illustrative instance is found in the Chardonnay grape, where white berries are suggested to result from a significant inversion and deletion, leading to hemizygosity at the MybA locus [1]. These examples motivate the need for a thorough understanding of SVs, which is vital for unraveling the diverse phenotypes observed in plants [10, 11]. However, identifying such variations relies on the availability of a high-quality reference genome.

The advent of high-throughput sequencing technologies has revolutionized the field of genomics, enabling the sequencing and assembly of high-quality plant genomes. These advancements have enabled the sequencing and assembly of high-quality plant genomes, including cassava. Cassava is a vital crop, especially for subsistence farmers in developing regions [12]. It serves as a source of nutrition for close to a billion people residing in the tropical regions of Africa, South America, and Asia [13]. Understanding cassava genetics is essential for addressing food security challenges and harnessing the crop’s potential for improvement. The release of several high-quality, chromosome-scale, haplotype-resolved genome assemblies of African cassava provides a powerful tool for exploring its genetic diversity, offering more profound insights into cassava genetics. Recently released genomes include TMEB117, TME204, and TME7 [14–16]. The TME204 and TMEB117 genomes were sequenced using Pacific Biosciences high-fidelity (HiFi) sequencing reads, while the TME7 genome was sequenced using the PacBio RSII system. HiFi sequence reads have proven to be of high quality and have produced highly accurate genomes [17]. These genomes have already established a high level of heterozygosity in cassava, and there is apparent substantial genetic variability between these cassava cultivars. In the study of the TME7 genome, a comparison was made with the cassava reference AM560-2 v6.1 genome [18].The TME7 genome revealed many SVs, with over 10,000 large SVs (50 − 10,000 bp) covering > 15.99 Mb of sequence. Additionally, > 5,000 large haplotype-specific SVs were discovered within the genome. These SVs in TME7 include insertions, deletions, tandem duplications, expansions, and contractions of repetitive elements. These findings show a substantial contribution of SVs to shaping the genomic landscape and potentially influencing phenotypic diversity in TME7 [16].

Our study focuses on whole-genome alignment using previously published cassava genomes of TMEB117, TME204, and AM560-2 v8.1 [19] to identify both haplotype-specific SVs and SVs across different cassava cultivars through comparative genomics. Haplotype-resolved assemblies are invaluable for delving into haplotype-specific structural variations within genomes and identifying genetic differences across other cassava genomes. Our results reveal structural diversity, including a large 9.7 Mbp insertion on chromosome 12 of TMEB117, absent in previously published genomes. This insertion is highly repetitive and contains unique genetic features, such as overrepresented transposable elements and genes associated with deacetylase activity. Understanding SVs, like this insertion, is crucial for comprehending the genetic landscape of cassava and its impact on phenotypic traits. This research provides new insights into cassava genetics, paving the way for developing improved cassava varieties with enhanced nutritional and agronomic characteristics.

## Results

### Large 9.7 mbp fragment on chromosome 12 of the TMEB117 genome

**Fig. 1:**
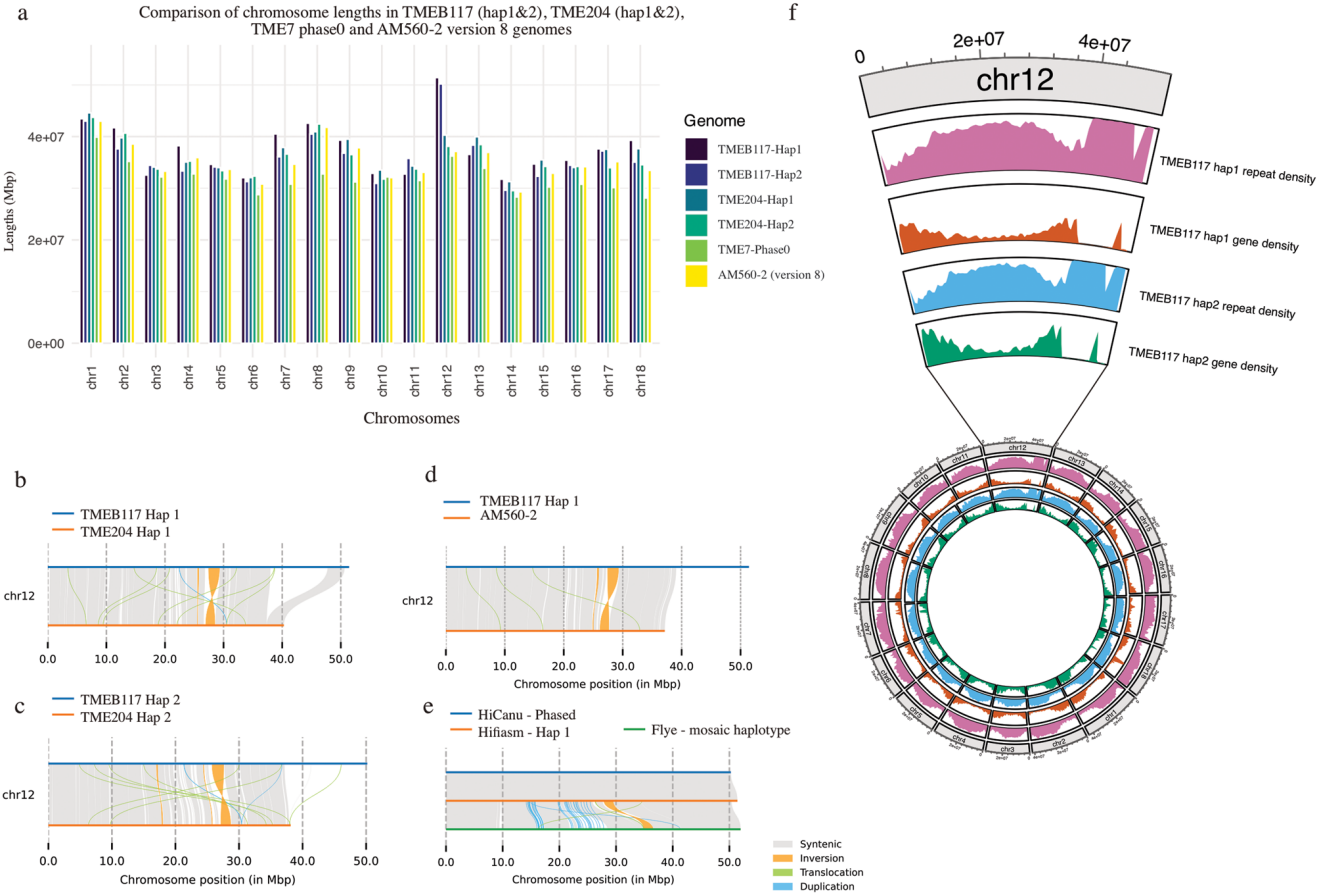
Chromosome lengths and pairwise comparison of chromosome 12 across cassava cultivars (**a**) Stacked bar plots display the chromosome lengths of TMEB117 (both haplotypes), TME204 (both haplotypes), TME7 (primary haplotype), and AM560-2 version 8 genome. The plot uncovers that TMEB117’s chromosome 12 exceeds 50 Mbp, exhibiting a considerable size difference compared to other cassava genome assemblies. (**b**, **c**) Pairwise synteny plots comparing chromosome 12 haplotypes (hap1 and hap2) of TMEB117 and TME204. (**d**) Comparison of TMEB117 chromosome 12 hap1 with the cassava reference genome AM560-2 (version 8). (**e**) Assembly of TMEB117 chromosome 12 using HiCanu (blue, primary haplotype), hifiasm (orange, hap1), and Flye (green, mosaic haplotype), (**f**) Zoomed in view of chromosome 12 from Landi et al., 2023, showing high repeat density and low gene density near the chromosome’s end.

Our recent study produced a high-quality haplotype-resolved genome for the TMEB117 cassava cultivar [14]. We examined this genome’s chromosome sizes and genomic features, as illustrated in (Fig. 1). Chromosome 12 in TMEB117 exhibits a larger size of approximately 51 Mbp, contrasting with the sizes observed in other cassava cultivars such as TME204 (40 Mbp), TME7 (36 Mbp), and AM560-2 (37 Mbp) (Fig. 1a). Conducting a pairwise alignment of chromosome 12 of TMEB117 with an African cultivar, TME204, and with the reference AM560-2 v8.1, we identified a large inserted fragment of 9.7 Mbp in the TMEB117 genome (Fig. 1b-d). 90% of the large insertion, located in a region characterized by low gene density and high repeat content in both haplotypes (Fig. 1f), is composed of transposable elements (TEs). Of these TEs, the terminal inverted repeats of the *MUDR-Mutator* superfamily accounted for 76% of the total annotated TEs in this region (Table 1). To identify TE superfamilies enriched in this insertion relative to the genome, we performed a Fisher’s exact test on the counts of TEs. This analysis uncovered significant enrichment for the *MUDR-Mutator*, Helitron, and CACTA superfamilies (Additional file 2: Table S2).

**Table 1:** The proportion of different classes of annotated transposons occupied in the hap1 and hap2 genomes at the insertion region (Coordinates: hap1 chr12:37986388–47748555; hap2 chr12:36042535–45804701)

Family	Superfamily	Total % - Hap 1	Total % - Hap2
TIR	MUDR-Mutator	76.30	76.5
LTR-RT	Gypsy	10.30	10.40
LTR-RT	Unknown	5.38	5.16
LTR-RT	Copia	0.82	0.85
Helitron	Helitron	0.08	0.16
TIR	CACTA	0.08	0.08
TIR	hAT	0.08	0.06
TIR	PIF-Harbinger	0.04	0.01
TIR	Tc1-Mariner	0.01	0.03
LINE	Unknown	0.02	0.03
MITE	CACTA	0.02	0
MITE	MUDR-Mutator	0.02	0.02
MITE	hAT	0.02	0.03
MITE	PIF-Harbinger	0.01	-

### Assembling TMEB117 chromosome 12 using three different assemblers (HiCanu, Flye, and hifiasm) to confirm the large insertion and resolve assembly errors

The unique genomic characteristic of chromosome 12 within TMEB117 prompted additional analysis to confirm the presence of this large fragment. We assembled the TMEB117 genome employing three different assembly tools: Hifiasm, HiCanu, and Flye [20–22]. Hifiasm was utilized to generate phased genomes of TMEB117. HiCanu produced a diploid genome that was then phased to a primary haplotype, and Flye produced a collapsed assembly, representing the diploid genome as a single mosaic haplotype. Both hifiasm and HiCanu assemblies showed better assembly statistics and completeness than Flye, with better contiguity, fewer contigs, and minimal duplication. While Flye achieved a higher N50, its genome size was overestimated (Additional file 1: Fig. S8; additional file 2: Table S6). The size of chromosome 12 from the phased HiCanu assembly was 50.26 Mbp, 51.42 Mbp for Hifiasm, and 51.98 Mbp for Flye. Synteny blocks spanning the entire chromosome 12 were observed while comparing the phased genomes obtained with HiCanu and Hifiasm assemblers, successfully capturing the whole insertion region in chromosome 12. However, the Flye assembly, which generated a mosaic haplotype genome, displayed duplications (Fig. 1e). Despite this, synteny was also observed towards the insertion region in the Flye assembly. Furthermore, pairwise alignment showed a lack of similarity between any portion of chromosome 12 sequences and other chromosomes in the genome, unplaced contigs, and the *Alternaria alternata* genome [23] (Additional file 1: Fig. S1), previously seen as a major contaminant.

### Coverage analysis of chromosome 12 with other cassava cultivars

The read mapping coverage analysis for chromosome 12 haplotypes across 16 cassava cultivars revealed several differences in read mapping patterns between cultivars (Additional file 1: Fig. S2). The mapped reads showed a high mean mapping quality (> 21) on both haplotypes (Additional file 2: Tables S1a and S1b). Uniform coverage within the insertion region across both haplotypes was observed in cultivars such as TME60444, COL2182, CUB40, TMEB117, TME3, TME7, TME14K, and TMS961089A indicating a high degree of similarity and the full-length insertion region in these cultivars with TMS961089A having the lowest and most uneven coverage profile (Additional file 1: Fig. S2 and Additional file 2: Table S1a & b). The read mapping patterns for the cassava reference genome AM560-2, Tree cassava (natural hybrid between *Manihot esculenta* and *Manihot glaziovii*), *Manihot esculenta subsp. flabellifolia* (FLA 496-1) and PER226 showed a slope towards the end of chromosome 12 of approximately 4 Mbp on hap1, with a similar pattern on hap2. However, on hap2, reads were mapped to the last portion of chromosome 12 after the slope on these cultivars (Additional file 1: Fig. S2– left panel). Even though these cultivars (AM560-2, FLA 469-1, PER226, and Tree cassava) had a drop of reads mapping towards the end of chromosome 12, reads covered the insertion regions within the specified coordinates of the insertion (Additional file 1: Fig. S2– right panel). Cultivars like TMEB419, TME204, and ECU41 displayed drops and peaks in read coverage within the insertion region, with a depression pattern. TMEB693 mapping coverage within the insert showed a clear depression of reads mapping in the insertion region. Additionally, hap2 of these cultivars (TMEB419, TME204, TMEB419, and ECU41) exhibited a slight slope of reads coverage at the end of chromosome 12. For ECU41, the read coverage showed a depression and a slope at the last portion of chromosome 12 on hap1. Meanwhile, hap2 showed a pattern similar to PER226, Tree cassava, FLA 496-1, and AM560-2, mapping reads to the last portion of the chromosome. While several South American cultivars (AM560-2, FLA 469-1, PER226, and Tree cassava) exhibited a noticeable drop in the read coverage toward the end of chromosome 12, this pattern was inconsistent across all studied cultivars. In contrast, African cultivars, whether they showed depressed read coverage at the insertion region or uniform coverage across chromosome 12, generally maintained consistent read mapping toward the end of chromosome 12.

### Unique read count analysis on gene features within the insertion

**Fig. 2:**
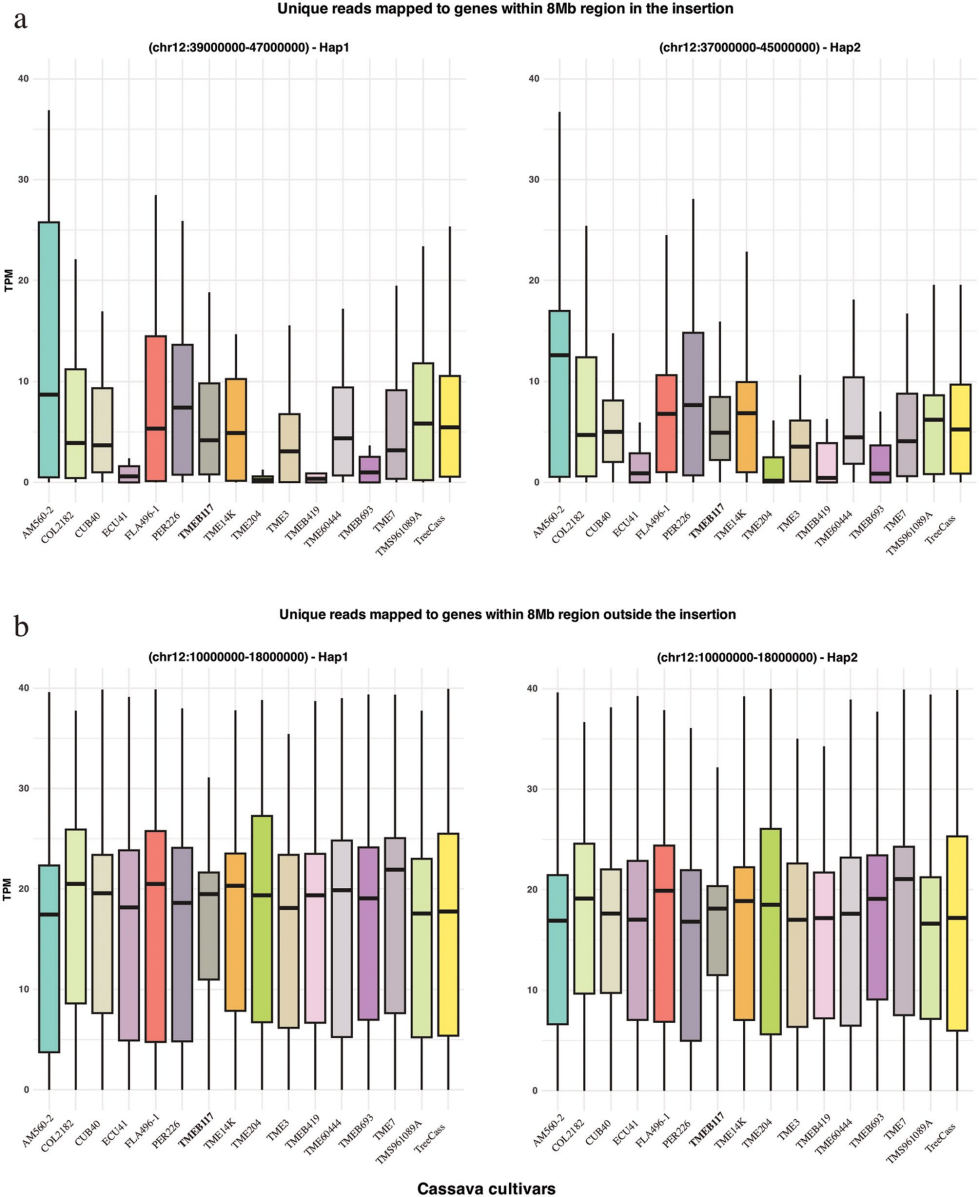
Boxplots of unique reads mapped to gene features within the 8 Mb insertion region and outside the inserted region. TMEB117 is highlighted in hold. (**a**) Normalized read counts (TPM) for gene features within the insertion region: chr12:39,000,000-47,000,000 on hap1 (left panel) and chr12:37,000,000-45,000,000 on hap2 (right panel). (**b**) Unique reads mapped to gene features outside the insertion region: chr12:1,000,000-18,000,000 on both haplotypes serving as a control.

Read coverage analysis provided insight into multiple mapped reads within a given genomic region, indicating the extent of sequencing depth even when only a single read is mapped at a specific position. We performed additional mapping count analysis, selecting only uniquely aligned reads within the 8 Mb insertion region (chr12:39,000,000–47,000,000 on hap1 and chr12:37,000,000–45,000,000 on hap2) and compared it to a control region 8 Mb away from the insert (chr12:1,000,000–18,000,000– same coordinates for both haplotypes). We analyzed 22 gene features on hap1 and 17 on hap2 within the insert region and 324 and 349 gene features outside the insert region on hap1 and hap2, respectively. Normalizing the read counts against the whole genome allowed for accurate comparison, accounting for differences in sequencing depth. Our analysis (Fig. 2) revealed that the AM560-2 cultivar showed the highest read counts on both haplotypes within the insertion region. Additionally, other genotypes also had higher mapping counts compared to TMEB117. Consistent with our coverage plot (Additional file 1: Fig. S2– right panel), cultivars such as TME204, TMEB419, ECU41, and TMEB693, which showed depression characteristics in read mapping, had the lowest average read counts. Of these, TMEB693 had slightly higher read counts in this region on hap1. These four cultivars had the lowest counts also on hap2, where the read counts were somewhat higher than those on hap1 (Fig. 2a). As expected, the average read counts across all cultivars outside the insertion region were similar on both haplotypes (Fig. 2b).

**Fig. 3:**
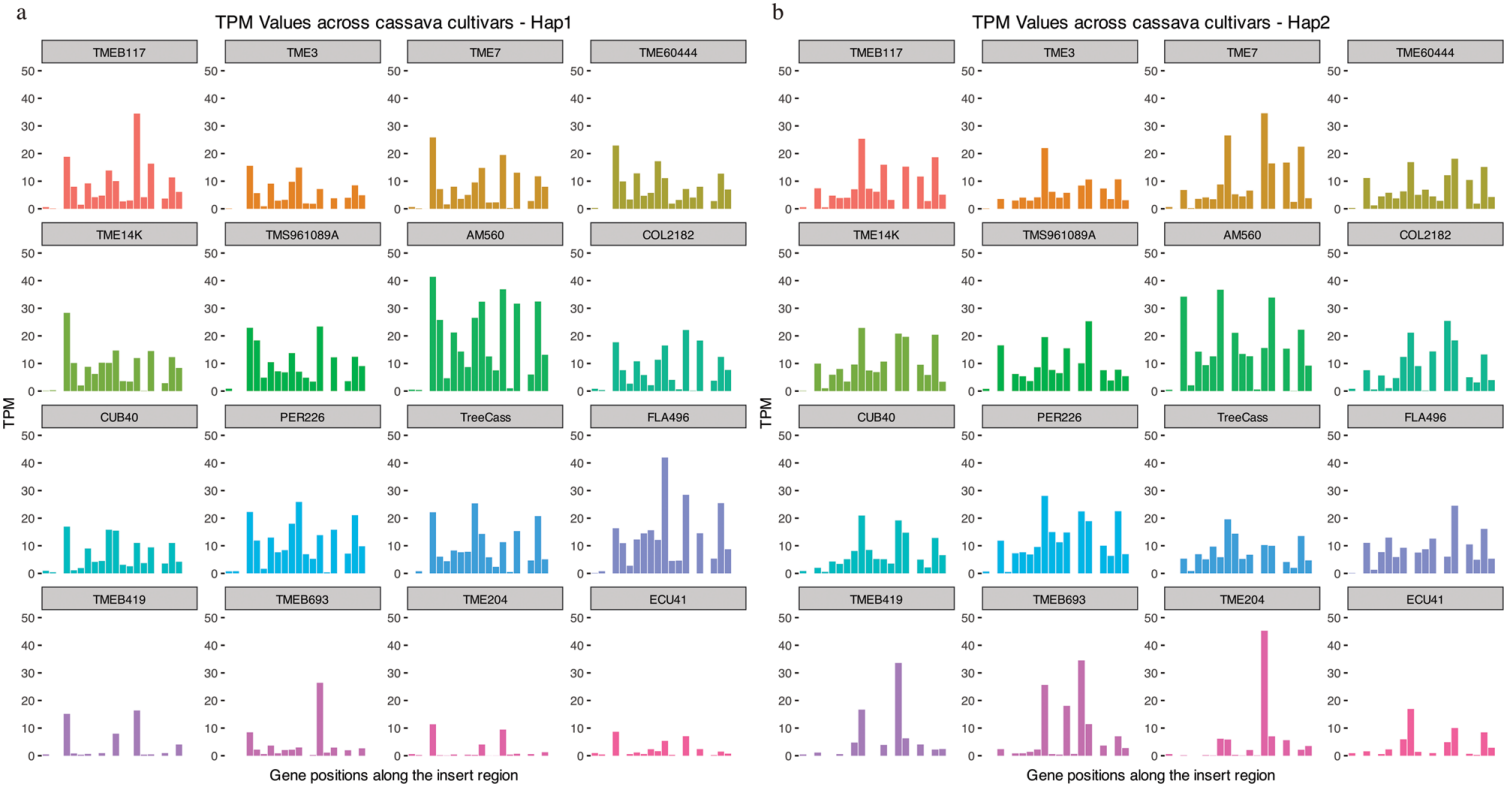
Gene-by-gene TPM values of read counts across all cultivars within the insertion region. (**a**) Distribution of TPM read count values for 22 gene features across all cultivars on hap1. (**b**) TPM read count values distributed for 17 gene features across all cultivars on hap2.

Further investigation into gene-by-gene read counts indicated that the AM560-2 cultivar had the highest read counts on both haplotypes (Fig. 3). Some gene features appeared repetitive due to lacking unique reads mapped across all cultivars. As already demonstrated above, TMEB419, TME693, TME204, and ECU41 had the lowest read counts, although certain gene features in these cultivars exhibited higher read counts (Fig. 3). Gene-by-gene plots on gene features outside the insertion region exhibited similar read counts across all cultivars (Additional file 1: Fig. S3).

### PCR confirms the presence of insertion region fragments in TMEB117 genomes

Following the mapping of unique reads, we observed high variability in read coverage within the insertion region across the cassava cultivars in this study. Based on the availability of plant material, we selected three cultivars - TMEB117, TMEB419, and TME693 - to confirm the presence of the insert region fragments. The repetitive nature of the insertion region made it challenging to obtain suitable primers. Nonetheless, using the TMEB117 hap1 insertion coordinates (chr12:37,986,388 − 47,748,555), we designed three unique primer sets to selectively amplify different fragments of the insertion region (Additional file 2: Table S3). We conducted multiplex PCR to verify the presence of these specific fragments in the cassava genomes of the TMEB117, TME693, and TMEB419 genotypes, incorporating the *Protein phosphatase 2 A* (PP2A) and *GTP-binding protein* (GTPb) positive control genes in the same reaction. We successfully amplified the insertion fragments A, B, and C (Additional file 2: Table S3) and obtained products of the correct sizes. (Additional file 1: Fig. S6) The results confirmed that all three fragments are present in the TMEB117 and are also detected in the TMEB693 and TMEB419 genotypes (Additional file 1: Fig. S6). The fragments amplified from the TMEB117 genome were sequenced to confirm the correct amplification.

### Gene enrichment analysis for the known genes in the insertion region

We observed the presence of 147 gene features in hap1 and 159 in hap2 by investigating insertion regions defined by specific coordinates (hap1 chr12:37986388–47748555; hap2 chr12:36042535–45804701). The hap1 insertion region had 37 known genes and 110 genes annotated as potential. Similarly, hap2 had 36 known genes and 123 potential genes. We ran a gene ontology (GO) enrichment analysis with the known genes from both haplotypes and identified significant gene sets. The GO analysis identified three significant enriched molecular function terms among the genes. The terms included “histone deacetylase activity” (GO:0004407), “protein lysine deacetylase activity” (GO:0033558), and “deacetylase activities” (GO:0019213) in both haplotypes. For each GO term, both haplotypes showed enrichment of two genes, HDA14 and SRT2, as indicated by the gene count of 2 (Additional file 2: Table S7a, b). To explain the unknown genes, BLAST searches were performed on the gene sequences extracted from the potential gene set for both haplotypes. The results revealed homologous sequences in *Manihot esculenta* chromosome 12, annotated with Manes IDs, and *Hevea brasiliensis* (Additional file 2: Table S4 and S5).

### Genome-wide comparative analysis across cassava cultivars

#### Large haplotypic structural variation within the TMEB117 genome

**Fig. 4:**
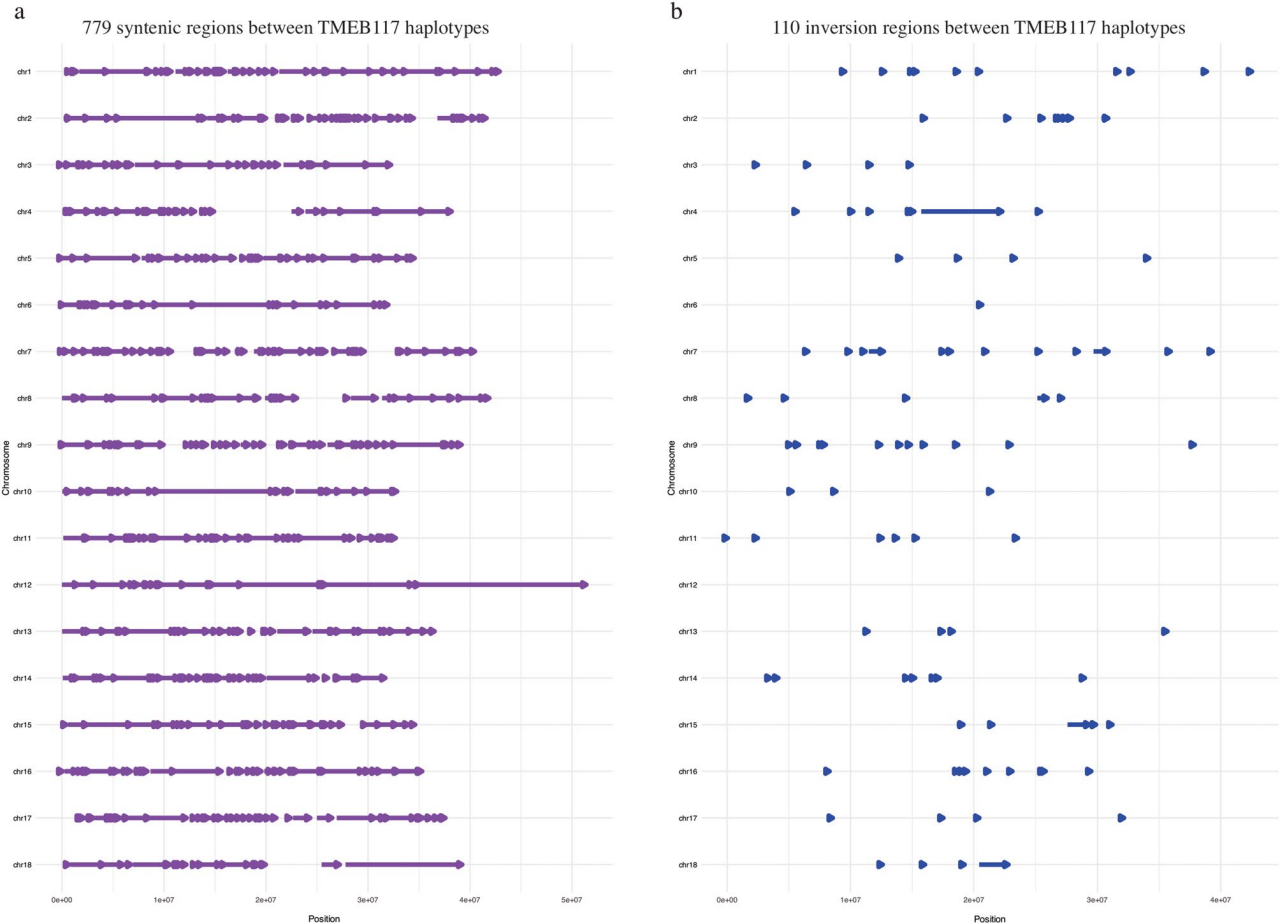
Syntenic and inversion regions within the TMEB117 genome across its chromosome. (a) Purple arrows between coordinates represent 779 syntenic regions per chromosome of TMEB117 haplotypes. (b) 110 inversion regions highlighted in blue arrows in TMEB117 haplotypes per chromosome.

We compared the two haplotypes to analyze differences within the TMEB117 genome. Our analysis showed unique structural variations specific to each haplotype. Structural annotations with predominant syntenic regions and observed large inversions are comprehensively illustrated (Fig. 4). A total of 779 syntenic regions were identified, spanning 551,743,349 bps (79.5%) in TMEB117 Hap1 and 547,212,985 bps (82.3%) in TMEB117 Hap2 (Fig. 4a). We identified 110 large inversions distributed across chromosomes. These inversions collectively span 18,588,121 bps (2.7%) in hap1 and 17,017,386 bps (2.6%) in hap2 genomes. Specifically, large inversions of more than 1Mbp were observed in chromosomes 4, 7, 15, and 18, with chromosome 4 having the largest inversion of 6.5Mbp in size (Fig. 4b). In addition to these variations, translocations, duplications, and non-aligned regions demonstrate the structural complexity of the TMEB117 haplotypes. The genome exhibited diverse variations, including 1,608,991 single nucleotide variants and 104 copy number variations, encompassing both copy gains and losses (Additional file 1: Fig. S4). Additionally, chromosome 12 had the highest average length of synteny blocks, measuring 2.1 Mbp (Additional file 1: Fig. S5). It also comprised the most extended synteny blocks in both haplotypes, with lengths of 16.3 Mbp and 16.2 Mbp (hap1 and hap2) towards the end of the chromosome (chr12:35034285–51416241 on hap1 and chr12:33191782–49458244 on hap2).

**Fig. 5:**
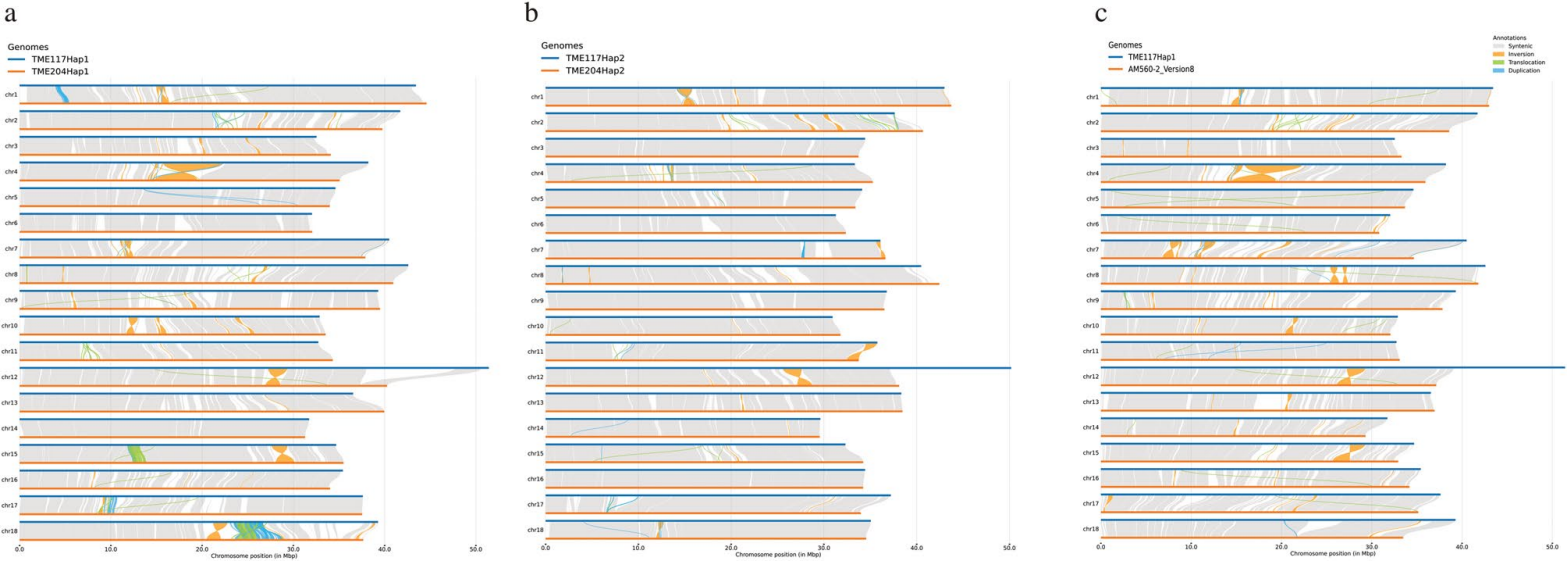
Synteny plots comparing haplotypes and genomes. (a) TMEB117 hap1 vs. TME204 hap1. (b) TMEB117 hap2 vs. TME204 hap2. (c) TMEB117 hap1 vs. AM560-2 v8. Blue and orange lines represent different genomes, with grey regions showing synteny and gaps indicating differences.

#### Comparative analysis of AM560-2 v8.1, TME204 and TMEB117 cassava genomes

As shown in (Fig. 1b), there is a notable observation of a huge 9.7 Mbp insertion region in chromosome 12. To inspect this finding and other structural variations, we conducted a comparative analysis with the whole genome of TME204 haplotypes and cassava reference genome AM560-2 v8.1 (Fig.5 ). The comparative synteny analysis uncovered distinctive genomic patterns, shedding light on structural variations among cassava cultivars. TMEB117 hap1 vs. TME204 hap1 and TMEB117 hap1 vs. AM560-2 v8.1 comparisons (Fig.5 a and c) revealed genomic signatures. The former showed 801 syntenic regions spanning 533,328,857 bps (76.9%) in TMEB117 hap1 and 527,567,427 bps (76%) in AM560-2 v8.1, accompanied by 105 inversions and 657 translocations. The latter unveiled 758 syntenic regions, covering 549,669,393 bps (79.2%) in TMEB117 hap1 and 551,866,350 bps (79.5%) in TME204 hap1, along with 96 inversions and 682 translocations. In the TMEB117 hap2 vs. TME204 hap2 comparison (Fig. 5b), 489 syntenic regions were identified, covering 578,212,474 bps (86.9%) in TMEB117 hap2 and 586,667,066 bps (83%) in TME204 hap2, 80 inversions, and 358 translocations.

## Discussion

Large structural variations observed among cassava genotypes emphasize the significant genomic diversity within the species. This study identified a major structural difference in chromosome 12 of the TMEB117 genome: 9.7 Mbp insertion region missing in earlier published cassava genomes. This insertion is characterized by a high repeat content and low gene density, reflecting the complexity of the cassava genome. We validated the presence of this insertion through multiple methods, including genome assembly using three tools (hifiasm, HiCanu, and Flye), pairwise alignment of chromosomal regions, and PCR confirmation of specific fragments. These methods consistently confirmed the insertion and ruled out the possibility of assembly artifacts due to misassembly or scaffolding. The insertion region comprises over 90% TEs, with a significant overrepresentation of the *MUDR-Mutator* TE superfamily [24, 25]. Transposable elements can influence gene regulation and genomic stability, and their abundance in this region may have important implications for genome function and evolution [26].

Comparative mapping coverage analysis of chromosome 12 haplotypes across 16 cassava cultivars using short-read sequencing revealed variability in the insertion region. Some cultivars, such as TME60444, COL2182, CUB40, TMEB117, TME3, TME7, and TME14K, displayed uniform read coverage across the insertion, indicating a conserved genomic structure. In contrast, other cultivars, including AM560-2, Tree cassava, FLA 496-1, and PER226, showed decreased read coverage toward the end of chromosome 12, suggesting potential structural differences. Compared to these, African landraces generally maintained consistent read mapping toward the end of chromosome 12, irrespective of whether they showed uniform or depressed coverage in the insertion region. This highlights the differences in genomic architecture between African and some South American cultivars at this locus. Although the insertion was absent in the final assemblies of some cultivars like AM560-2 and TME7 (Fig. 5a-c, S7), raw data indicated that reads cover the entire region, unlike TME204, which lacked coverage entirely. This suggests difficulties assembling repetitive sequences.

Further analysis of uniquely mapped reads provided more detailed insights into the insertion region’s gene features and variability. AM560-2 had a higher read count within the insertion region than other cultivars. However, the sequencing data indicated fewer unique reads in TMEB693, TME204, TMEB419, and ECU41 cultivars. PCR analysis confirmed the presence of fragments of the insertion region in TMEB693 and TMEB419. Due to the non-quantitative nature of the PCR analysis, we could not confirm the lower read counts found by the unique read count analysis in TMEB693 and TMEB419. In fact, in these PCR reactions, the amplification of detectable regions reaches saturation independently of the template amount used. This discrepancy between the sequencing depth and PCR amplification is due to PCR’s sensitivity to low-abundance sequences, leading to saturation. To clarify these differences, future studies should employ quantitative PCR (qPCR) [27] to measure the copy number of the insertion sequences accurately. This approach would provide precise quantification and help resolve inconsistencies between sequencing and PCR results, leading to a clearer understanding of the genomic structure of the region.

Our analysis revealed that although no genotype completely lacks the insertion, many exhibit partial coverage. The variability in read mapping patterns suggests that this insertion is a highly polymorphic region, possibly driven by its repetitive sequence content, which may promote recombination events and structural changes. This defines the insertion as a hypervariable [28] locus contributing to cassava cultivars’ overall genomic diversity. Future studies should incorporate long-read sequencing technologies across diverse cassava genotypes to better understand this region’s complexity. Such approaches could provide more accurate assemblies of highly repetitive regions and help clarify the structural variation observed in this insertion.

Gene ontology enrichment analysis shows two significant unique genes, HDA14 and SRT2, within the genome’s insertion region. HDA14, as a histone deacetylase, contributes to removing acetyl groups from histone proteins, promoting a closed chromatin conformation [29]. On the other hand, SRT2, a member of the sirtuin family, also possesses deacetylase activity targeting acetylated proteins, including histones, to regulate chromatin structure by removing acetyl groups from lysine residues on histones [30]. Histone deacetylases induce the deacetylation of histone lysine residues, forming a tighter chromatin structure. The presence of these genes in the insertion region could imply their potential role in influencing the structural arrangement of the genome. Their roles in chromatin modification and plant epigenetics regulations may influence the inserted region’s stability and organization. Further exploration of their specific interactions and downstream effects within the insertion region could disclose unknown insights into the functional consequences of large structural variations in the genome.

A genome-wide comparison of the TMEB117 cassava genome with other cultivars revealed extensive structural variation, underlining the complexity and diversity within cassava genomes. Structural variations represent a large component of genetic diversity within the genomes of eukaryotic organisms and can potentially impact the organism’s fitness [31]. Our synteny analysis of the TMEB117 cassava genome revealed haplotype-specific structural variations, including large inversions, translocations, and duplications. These findings highlight the structural complexity of the TMEB117 genome. Comparative analysis with other cassava genomes, such as AM560-2 and TME204, further underlines the structural diversity among cultivars. The large 9.7 Mbp insertion on chromosome 12 in TMEB117, absent in the final assemblies of other cultivars but present in raw reads, suggests that such structural variants could be more common in cassava than previously recognized. This large insertion and extensive synteny blocks within this region, consistent across both haplotypes and homologous sequences of the TMEB117 genome (Fig.4 a), also confirm that these are genuine genomic features rather than assembly artifacts.

## Conclusions

While this study does not explore the detailed implications of the identified large insertion and other SVs, it does confirm the presence of the large insertion. These findings are a step towards exploring the potential impact of SVs, highlighting the genetic complexity within plant genomes and emphasizing the importance of delving deeper into structural variations to unravel the complexities of genetic diversity and evolutionary processes. The large insertion can be considered a valuable resource for pan-genome analysis of the cassava genome, offering insights into its unique features, primarily its high degree of heterozygosity. Additionally, possible analyses with different traits, such as genome-wide association studies at the SV level, could further explain the functional impact of this region on various phenotypes. A comprehensive understanding of the landscape of SVs offers confidence in uncovering the mechanisms underlying plant adaptation, resilience, tolerance to pathogens, and agronomic traits, guiding informed breeding strategies and crop improvement efforts.

## Methods

### DNA extraction for sequencing of TMEB117 and TMEB693

To prepare the DNA library, we extracted genomic DNA (gDNA) from expanded leaves of the genotypes TMEB117 and TMEB693 using the NucleoBond^®^ High Molecular Weight DNA kit, following the manufacturer’s instructions. We modified the protocol by extending the incubation at 50 °C to 45 min, dramatically increasing the final yield. The extracted gDNA was then sent for library preparation and subsequent sequencing.

### DNA extraction for PCR

gDNA was extracted from leaves of TMEB117, TMEB419 and TMEB693 cassava plants by combining the cetyltrimethylammonium bromide (CTAB) extraction method and spin-column‐based purification methods. Total nucleic acid was extracted by using the CTAB (2% CTAB,2% PVP‐40, 20 mM Tris–HCl, pH 8.0, 1.4 M NaCl, 20 mM ethylene-diaminetetraacetic acid) extraction protocol as described by Li et al. [32] with some modifications. Approximately 500 mg samples were ground in liquid nitrogen and mixed with 1 ml pre‐heated CTAB extraction buffer. Samples were incubated at 65 °C for 15 min with intermittent vortexing, then subjected to centrifugation (16 000 g at 4 °C for 5 min). The supernatant was mixed with an equal volume of cold chloroform: isoamyl alcohol (24:1) before centrifugation (16 000 g at 4 °C for 10 min). The supernatant was added to 0.6 volumes of cold isopropanol and gently mixed to precipitate the nucleic acids. The pellet was washed with 70% ethanol, air‐dried, and dissolved in nuclease‐free water. After RNaseA treatment, the resulting gDNA was cleaned using the kit DNA Clean & Concentrator™ (Zymo Research) according to the manufacturer’s instructions. The gDNA was then sequenced.

### PCR validation of the insertion region

The coordinates for the start and end points of the insertion regions in both genome haplotypes were derived using the SyRi (version– 1 0.6.3) [33] outputs obtained from the minimap2 (version– 2.26-r1175) alignments between TMEB117 and TME204 haplotypes. We extracted all the insertion regions labeled ‘INS’ on chromosome 12 and got the coordinates for the largest insertion within this specific chromosome for both haplotypes. Using hap1 insertion coordinates, we designed primers using the primer3 [34] online tool to get a unique specific primer set that could amplify fragments within the region. It is important to note that these coordinates are based solely on the pairwise alignment of TMEB117 and TME204, and therefore, the exact coordinates of the insertion region may differ in other cultivars.

PCR amplification was conducted in 20 µl of PCR mixture containing 20ng of cassava gDNA, 1x GoTaq Buffer with 1.5 mM MgCl_2_, 0.04 mM of dNTP, 0.4 µM of each primer, and 0.25 units of GoTaq DNA polymerase (Promega). The thermal cycling conditions were initial denaturation at 94 °C for 1 min, 35 cycles of 30 s at 94 °C, 30 s at 58 °C, and 2 min at 72 °C, and final extension at 72 °C for 5 min. The PCR primers used in this study are shown in Additional file 2: Table S3.

### Assembling TMEB117 chromosome 12 using three different assemblers (HiCanu, Flye, and hifiasm)

The TMEB117 genome was assembled using HiCanu (version– 2.3) Flye (version– 2.9.3-b1797). HiCanu is a diploid-aware assembler that produces diploid genomes. In contrast, the Flye assembler generated a single “collapsed” assembly represented a mosaic of both haplotypes. HiCanu’s diploid genome was further separated using Purge Haplotigs [35], resulting in a phased-diploid assembly. Hifiasm (version– 0.16.1-r375) haplotype-resolved TMEB117 genome, previously published, was also utilized. BUSCO (version– 5.3.2) and QUAST (version– 5.1) analyses were performed to assess assembly metrics, and these assemblies were used to validate the presence of the insertion region identified through comparative analysis. We aligned chromosome 12 assemblies of the three assembly tools using minimap2 [36], visualized the alignment using plotsr (version– 1.1.2) [37]. Since we have two haplotypes from the hifiasm assembly. Hap1 was compared against the primary assemblies of HiCanu and Flye assemblers to confirm that sequences of the haplotypes are represented within the insertion region. In addition, we compared using dot plots TMEB117 hap1 chromosome 12 to all TMEB117 hap1 chromosomes, unplaced contigs, and *Alternaria alternata* genome [23] to confirm that the existence of the insertion was not a result of assembly error.

### Coverage analysis of chromosome 12 with other cassava cultivars

To compare coverage across other cassava genotypes, we collected raw short-read sequences of 13 cassava genotypes from previously published data for our study. These genotypes include wild-type subspecies FLA 496-1 (*Manihot esculenta ssp. fabellifolia*), Tree cassava, a natural hybrid between *Manihot esculenta* and *Manihot glaziovii*, five South American (AM560-2, CUB40, ECU41, COL2182, and PER226), and five Tropical *Manihot esculenta* genotypes (TME204, TME3, TME60444, TME7, and TME14K), one Tropical Manihot Selection (TMS961089A) [15, 16, 19, 38]. We sequenced Illumina short-reads for the TMEB117, TMEB693, and TMEB419 and mapped all the 16 cassava cultivars to the whole genome haplotypes using Burrows-Wheeler Alignment tool (BWA) (version– 0.7.13-r1126) with default parameters [39]. We then extracted the alignment of chromosome 12. We used the mapped short-read data to investigate the coverage of the insertion region and the entirety of chromosome 12 in the TMEB117 genotype using samtools (version– 1.15.1) [40] (parameters: coverage -m, default bin size 40 ). The coverage is the percentage of positions within a given window with at least one aligned read. Samtools calculates this by computing the number of bases that align to each position within the region and generating a histogram (Additional file 1: Fig. S2).

### Read count analysis on gene features within the insertion

In addition to the coverage analysis, we investigated unique reads mapping to gene features within two regions: an 8 Mbp insertion region (chr12:39,000,000–47,000,000 on hap1 and chr12:37,000,000–45,000,000 on hap2) and an 8 Map control region outside the insertion region (chr12:1,000,000–18,000,000). We selected the 8 Mbp length to avoid the borders of the 9.7 Mbp insertion. We maintained a 1 Mbp buffer on either side to prevent including sequences too close to the boundaries, which might be less reliable. Using BWA alignment from coverage analysis, we extracted unique reads mapping the whole genome by excluding alternative and supplementary alignments and filtering out reads with a mapping quality of 0. We systematically analyzed unique mapping reads per gene for each cultivar. First, we extracted the unique mapping reads per gene from BAM files using the featureCounts tool (version– 2.0.6) [41], resulting in a BED file with chromosome, start, end, and count for each gene. Next, we calculated each gene’s Reads Per Kilobase (RPK). We then computed a genome-wide scaling factor by summing the RPK values of all genes. We used this factor to normalize the RPK per gene, yielding Transcripts Per Million (TPM), ensuring the total TPM values are summed to a million. Finally, we extracted genes from the two specific regions to compare their TPM values across all cultivars.

### Gene enrichment analysis

The genomic gene features were obtained from both haplotypes of the TMEB117 genome on chromosome 12. We extracted all genes in the insertion region, distinguishing known genes from potential ones to enable further examination of function. The known genes within the insertion region underwent a gene ontology (GO) enrichment analysis using the Arabidopsis thaliana database through R’s enrichGo function of the clusterProfiler package (version– 4.10.1) [42]. The gene IDs were reformatted to ensure compatibility with the database, and the resulting set of genes underwent enrichment analysis to reveal potential associations with their functions. A BLAST search was also conducted for the potential genes from our annotations. The BLAST outputs were filtered based on percentage identity, query coverage (> 90%), and e-value (< 1e-05). The resulting RefSeq IDs from the filtered blast outputs were used as inputs for the Entrez database [43] to determine the functions of these genes.

### Enrichment analysis of transposable elements superfamilies

Fisher’s exact test was performed to determine whether specific transposable element (TE) superfamilies were overrepresented in the insertion region of chromosome 12 compared to the whole genome TE annotations. The null hypothesis assumed a random distribution of TEs across the genome, with no specific enrichment in the insertion region. For each TE superfamily, a contingency table was generated to compare the counts of TEs within the insertion region against those in the rest of the genome. The contingency table for each superfamily was constructed as follows: the number of TEs of the superfamily within the insertion region, the total number of TEs in the insertion region minus the count of the superfamily’s TEs, the number of TEs of the superfamily in the genome, and the total number of TEs in the genome minus the count of the superfamily’s TEs. Fisher’s exact test was then applied to each contingency table to assess the statistical significance of enrichment. The resulting p-values were adjusted using the Benjamini-Hochberg method to account for multiple tests.

### Genome-wide comparative analysis across cassava cultivars

Plotsr [37] was employed for visualizing the genome-wide comparisons between TMEB117 haplotypes (hap1 and hap2), followed by comparisons of these haplotypes with three other genomes - AM560-2 v8.1 and TME204 cassava cultivars. Plotsr utilizes minimap2 and SyRI outputs for this analysis. Minimap2 is used for whole-genome alignment, and SyRI [33] is used to identify conserved synteny and structural rearrangements across the genomes. The outputs of SyRI provided insights into the extent of structural variations observed within the TMEB117 genome and across different cassava cultivars.

## Electronic supplementary material

Below is the link to the electronic supplementary material.

Supplementary Material 1

Supplementary Material 2

## Data Availability

Raw Illumina reads for TMEB117, TMEB693, and TMEB419 can be accessed from the National Center for Biotechnology Information (NCBI) Sequence Read Archive (SRA) database under BioProject PRJNA1149535, with accession numbers SRR30294079, SRR30294078 and SRR30294077. Additionally, data for other cassava cultivars reused in this study are available under the following accessions: Tree cassava (SRR2847469), AM560-2 (SRR2847385), TME60444 (SRR2847379), FLA 496-1 (SRR2847408), TME3 (SRR1261610), TME7 (SRR16021941), TME14K (SRR2847461) and TME204 (ERR5484651). Stephan Winter’s team (Leibniz Institute DSMZ) provided data for South American cultivars (CUB40, ECU41, COL2182, and PER226). Ramu Punna from Cornell University provided data for the TMS961089A genotype. These data are currently restricted to this manuscript and will be available once all teams involved complete their analysis.
