# Supplementary material for: Genome-wide comparison reveals large structural variants in cassava landraces
Source: BMC Genomics. 2025 Apr 10;26:362. doi: 10.1186/s12864-025-11523-y (PMC11987339; doi:10.1186/s12864-025-11523-y)
Supplement: Supplementary file 1 — Supplementary Material 1 [file 12864_2025_11523_MOESM1_ESM.pdf]

Genome-wide comparison reveals large structural variants in the cassava landraces

Supplementary Figures

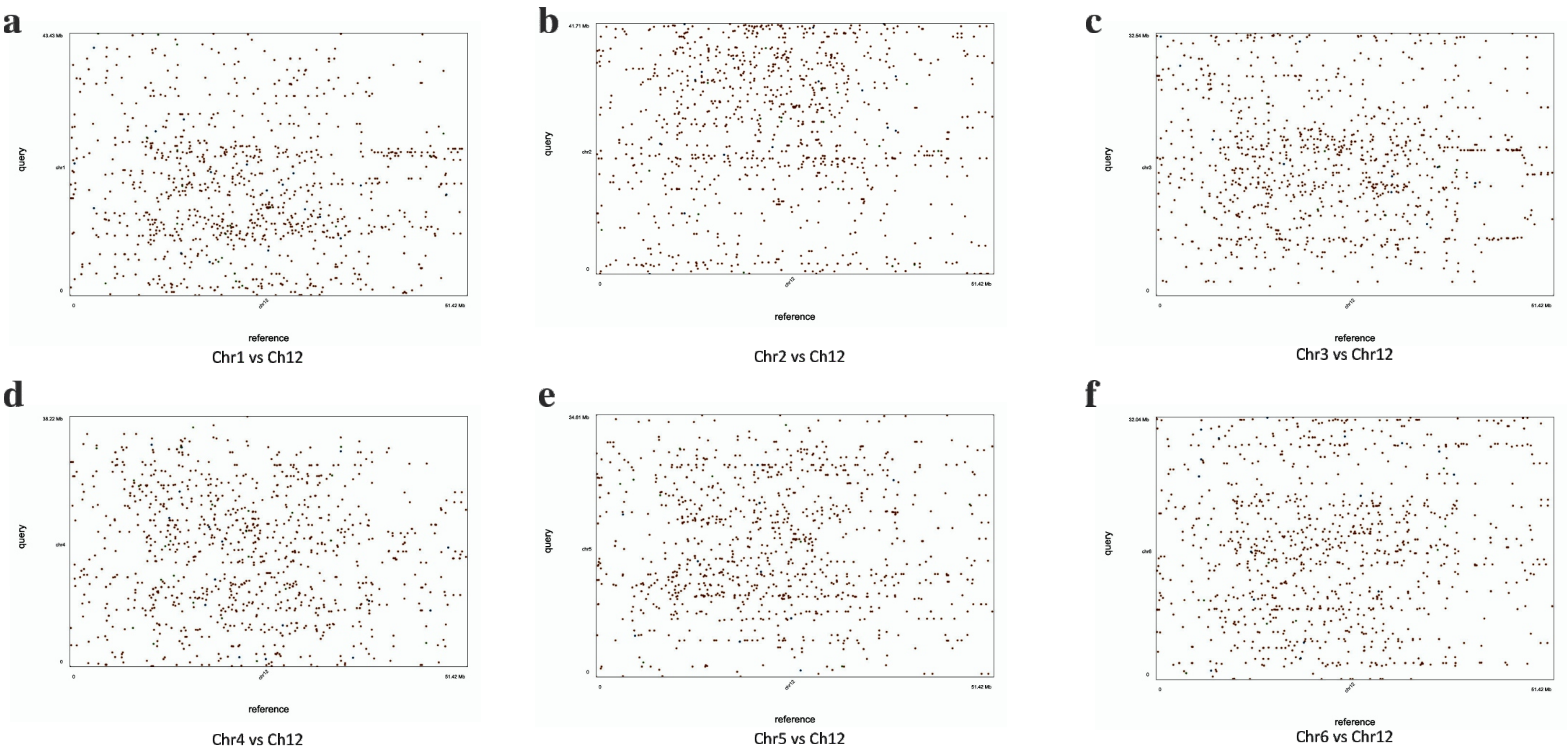

Chr1 vs Ch12

Chr2 vs Ch12

Chr3 vs Chr12

Chr4 vs Ch12

Chr5 vs Ch12

Chr6 vs Chr12

g

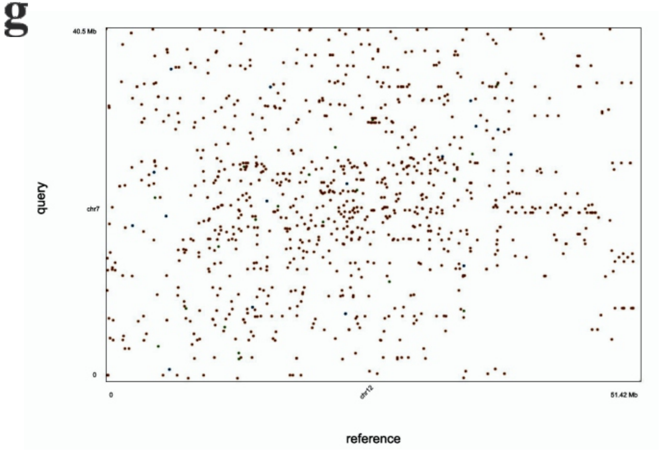

Chr7 vs Ch12

h

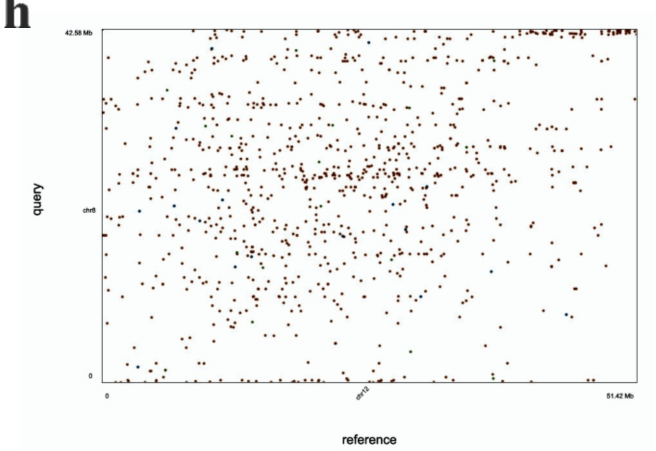

Chr8 vs Ch12

I

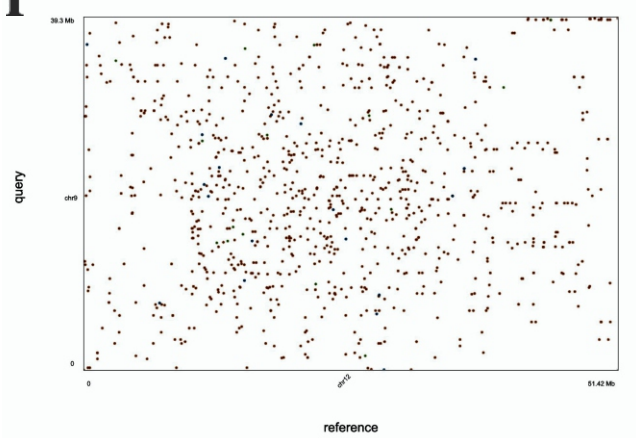

Chr9 vs Chr12

j

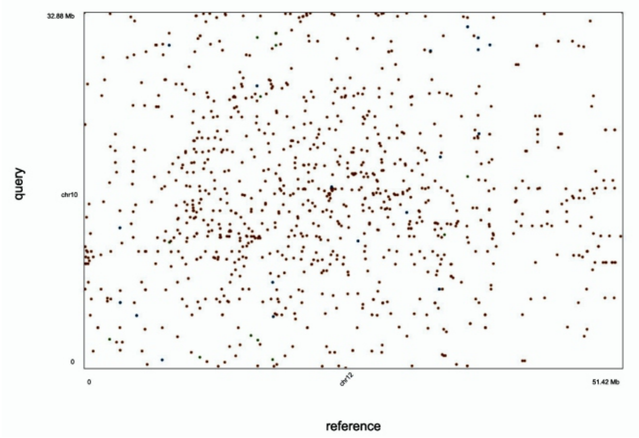

Chr10 vs Ch12

k

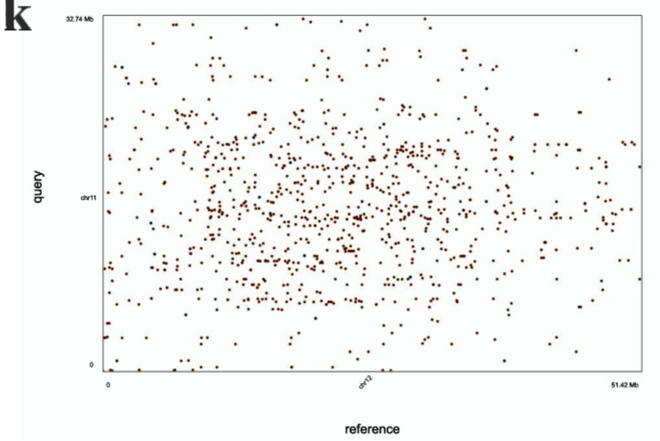

Chr11 vs Ch12

l

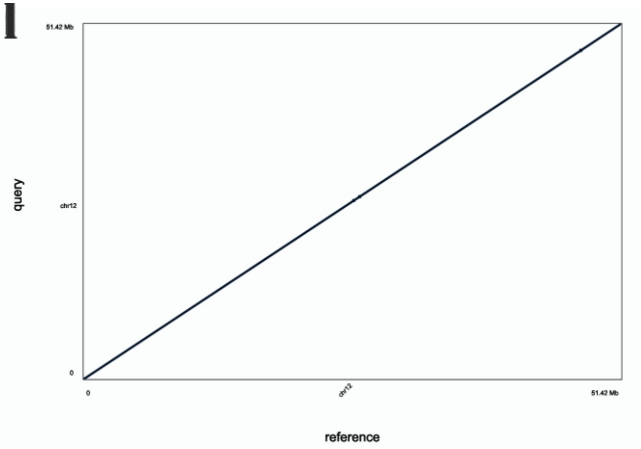

Chr12 vs Chr12

m

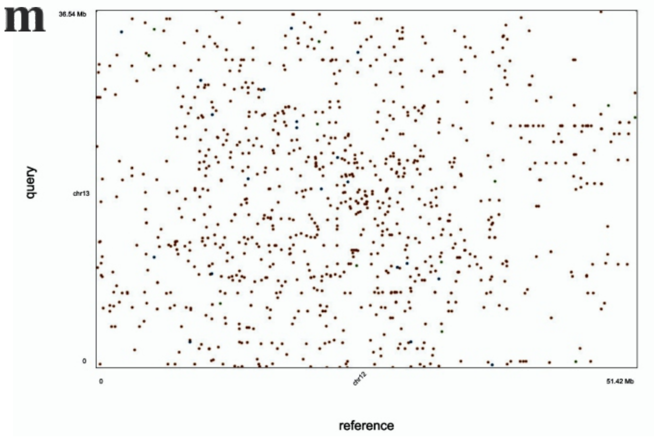

Chr13 vs Ch12

n

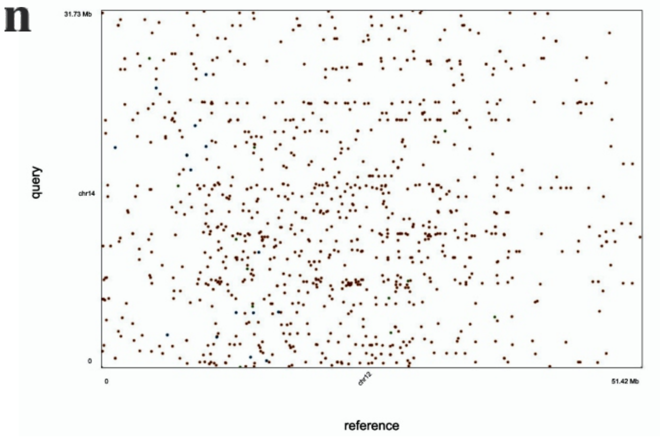

Chr14 vs Ch12

o

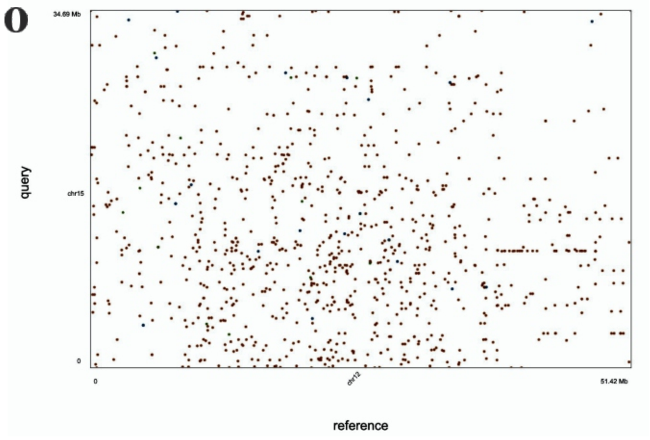

Chr15 vs Chr12

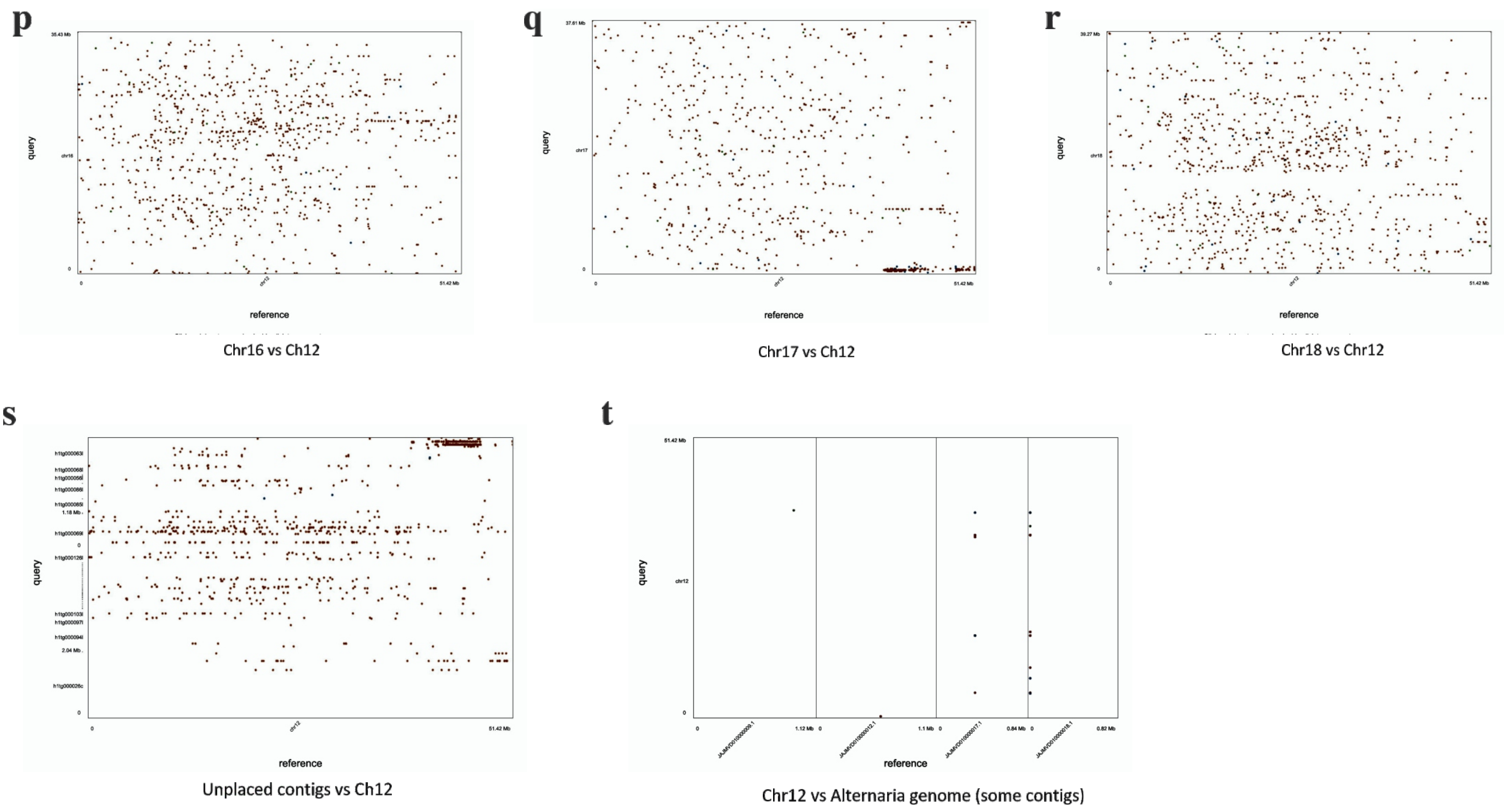

Fig. S1: Dot plots showing pairwise alignment of TMEB117 chromosome 12 with other chromosomes, unplaced contigs, and the Alternaria alternata genome

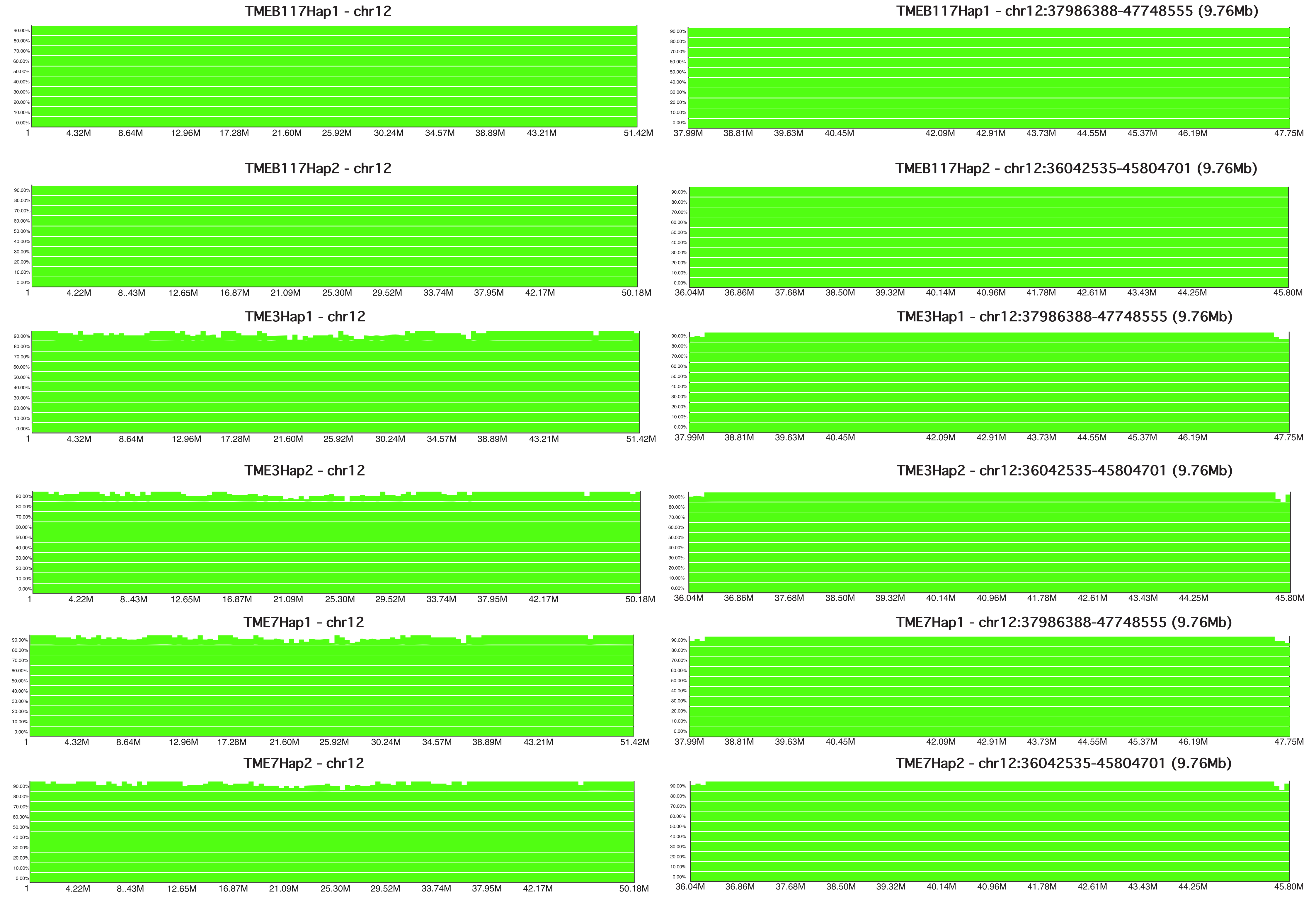
TMEB117Hap1 - chr12
TMEB117Hap1 - chr12:37986388-47748555 (9.76Mb)
TMEB117Hap2 - chr12
TMEB117Hap2 - chr12:36042535-45804701 (9.76Mb)
TME3Hap1 - chr12
TME3Hap1 - chr12:37986388-47748555 (9.76Mb)
TME3Hap2 - chr12
TME3Hap2 - chr12:36042535-45804701 (9.76Mb)
TME7Hap1 - chr12
TME7Hap1 - chr12:37986388-47748555 (9.76Mb)
TME7Hap2 - chr12
TME7Hap2 - chr12:36042535-45804701 (9.76Mb)
0.00% 10.00% 20.00% 30.00% 40.00% 50.00% 60.00% 70.00% 80.00% 90.00%
1 4.32M 8.64M 12.96M 17.28M 21.60M 25.92M 30.24M 34.57M 38.89M 43.21M 51.42M
1 4.22M 8..43M 12.65M 16.87M 21.09M 25.30M 29.52M 33.74M 37.95M 42.17M 50.18M
37.99M 38.81M 39.63M 40.45M 42.09M 42.91M 43.73M 44.55M 45.37M 46.19M 47.75M
36.04M 36.86M 37.68M 38.50M 39.32M 40.14M 40.96M 41.78M 42.61M 43.43M 44.25M 45.80M

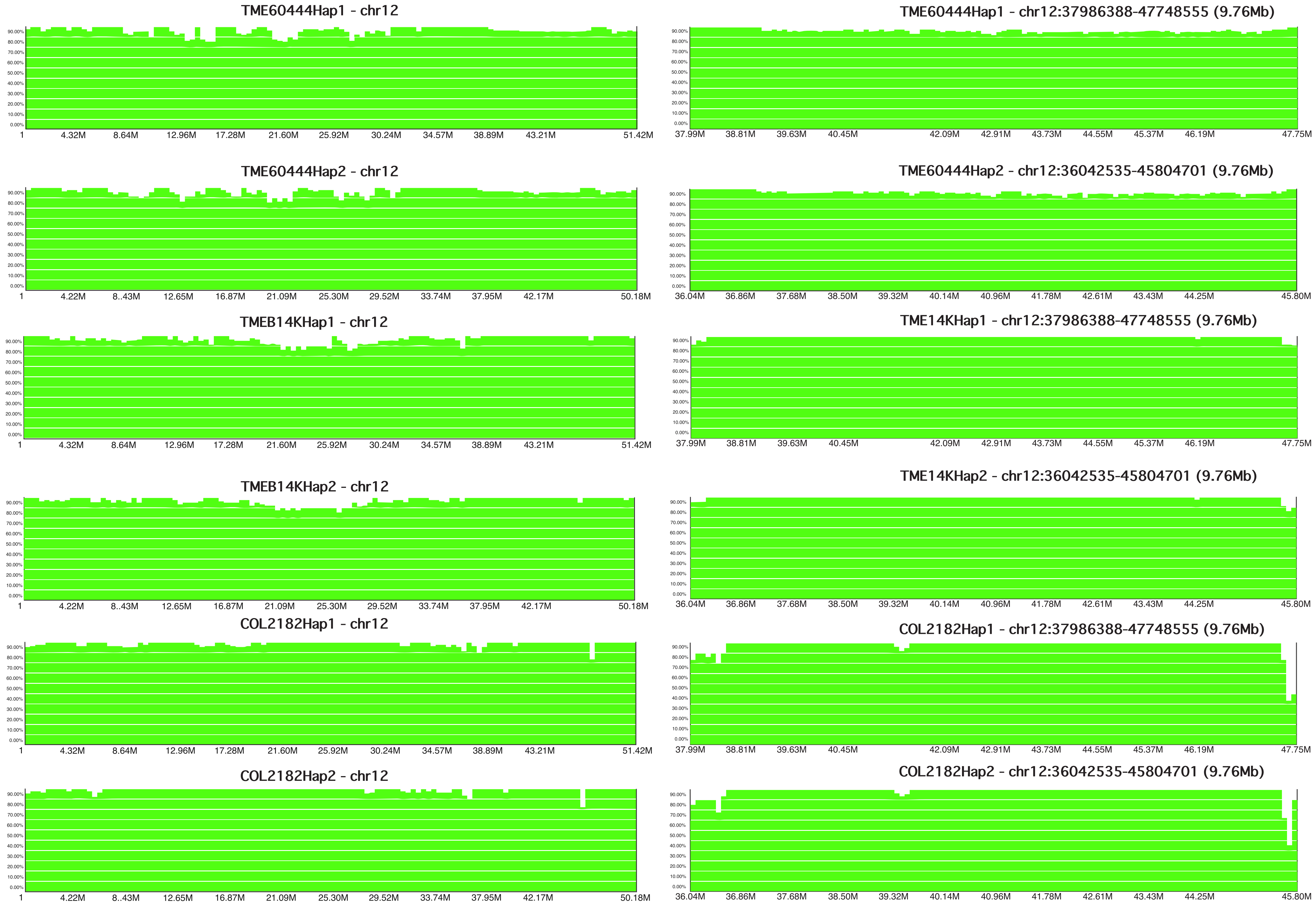

TME60444Hap1 - chr12
1 4.32M 8.64M 12.96M 17.28M 21.60M 25.92M 30.24M 34.57M 38.89M 43.21M 51.42M
TME60444Hap1 - chr12:37986388-47748555 (9.76Mb)
37.99M 38.81M 39.63M 40.45M 42.09M 42.91M 43.73M 44.55M 45.37M 46.19M 47.75M
TME60444Hap2 - chr12
1 4.22M 8..43M 12.65M 16.87M 21.09M 25.30M 29.52M 33.74M 37.95M 42.17M 50.18M
TME60444Hap2 - chr12:36042535-45804701 (9.76Mb)
36.04M 36.86M 37.68M 38.50M 39.32M 40.14M 40.96M 41.78M 42.61M 43.43M 44.25M 45.80M
TMEB14KHap1 - chr12
1 4.32M 8.64M 12.96M 17.28M 21.60M 25.92M 30.24M 34.57M 38.89M 43.21M 51.42M
TME14KHap1 - chr12:37986388-47748555 (9.76Mb)
37.99M 38.81M 39.63M 40.45M 42.09M 42.91M 43.73M 44.55M 45.37M 46.19M 47.75M
TMEB14KHap2 - chr12
1 4.22M 8..43M 12.65M 16.87M 21.09M 25.30M 29.52M 33.74M 37.95M 42.17M 50.18M
TME14KHap2 - chr12:36042535-45804701 (9.76Mb)
36.04M 36.86M 37.68M 38.50M 39.32M 40.14M 40.96M 41.78M 42.61M 43.43M 44.25M 45.80M
COL2182Hap1 - chr12
1 4.32M 8.64M 12.96M 17.28M 21.60M 25.92M 30.24M 34.57M 38.89M 43.21M 51.42M
COL2182Hap1 - chr12:37986388-47748555 (9.76Mb)
37.99M 38.81M 39.63M 40.45M 42.09M 42.91M 43.73M 44.55M 45.37M 46.19M 47.75M
COL2182Hap2 - chr12
1 4.22M 8..43M 12.65M 16.87M 21.09M 25.30M 29.52M 33.74M 37.95M 42.17M 50.18M
COL2182Hap2 - chr12:36042535-45804701 (9.76Mb)
36.04M 36.86M 37.68M 38.50M 39.32M 40.14M 40.96M 41.78M 42.61M 43.43M 44.25M 45.80M
90.00% 80.00% 70.00% 60.00% 50.00% 40.00% 30.00% 20.00% 10.00% 0.00%

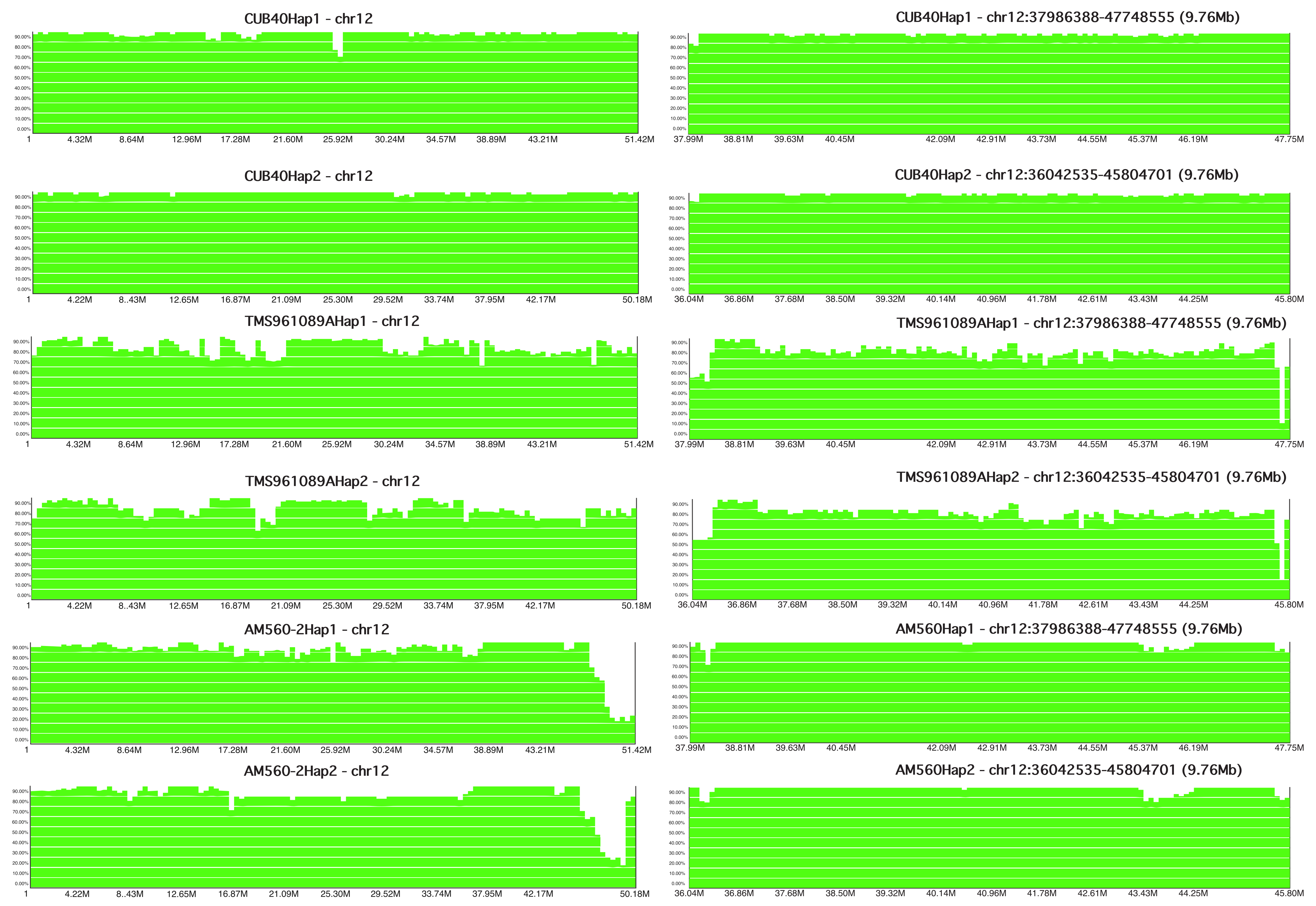
CUB40Hap1 - chr12
CUB40Hap1 - chr12:37986388-47748555 (9.76Mb)
CUB40Hap2 - chr12
CUB40Hap2 - chr12:36042535-45804701 (9.76Mb)
TMS961089AHap1 - chr12
TMS961089AHap1 - chr12:37986388-47748555 (9.76Mb)
TMS961089AHap2 - chr12
TMS961089AHap2 - chr12:36042535-45804701 (9.76Mb)
AM560-2Hap1 - chr12
AM560Hap1 - chr12:37986388-47748555 (9.76Mb)
AM560-2Hap2 - chr12
AM560Hap2 - chr12:36042535-45804701 (9.76Mb)
1 4.32M 8.64M 12.96M 17.28M 21.60M 25.92M 30.24M 34.57M 38.89M 43.21M 51.42M
37.99M 38.81M 39.63M 40.45M 42.09M 42.91M 43.73M 44.55M 45.37M 46.19M 47.75M
1 4.22M 8..43M 12.65M 16.87M 21.09M 25.30M 29.52M 33.74M 37.95M 42.17M 50.18M
36.04M 36.86M 37.68M 38.50M 39.32M 40.14M 40.96M 41.78M 42.61M 43.43M 44.25M 45.80M

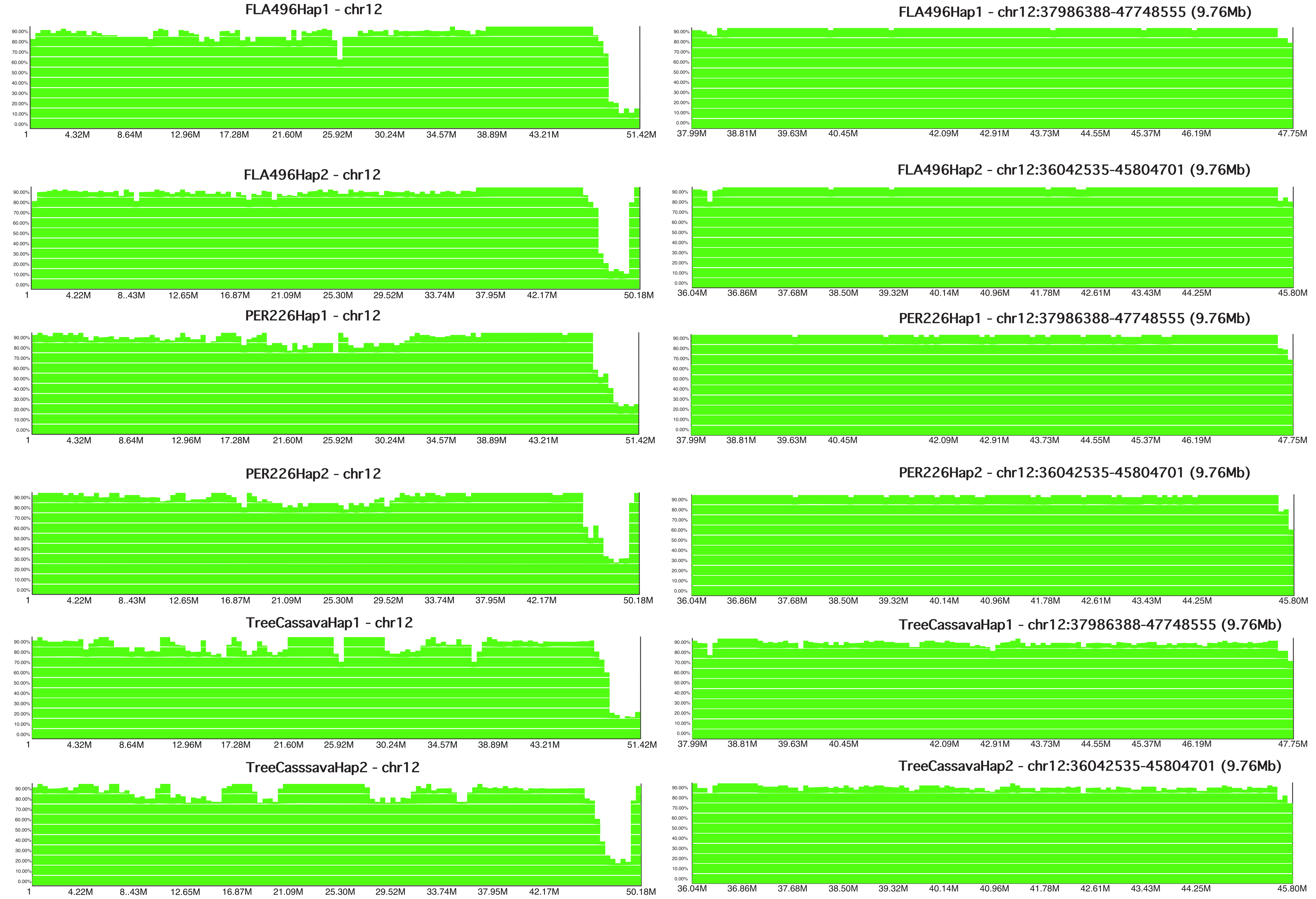
FLA496Hap1 - chr12
1 4.32M 8.64M 12.96M 17.28M 21.60M 25.92M 30.24M 34.57M 38.89M 43.21M 51.42M
FLA496Hap1 - chr12:37986388-47748555 (9.76Mb)
37.99M 38.81M 39.63M 40.45M 42.09M 42.91M 43.73M 44.55M 45.37M 46.19M 47.75M
FLA496Hap2 - chr12
1 4.22M 8..43M 12.65M 16.87M 21.09M 25.30M 29.52M 33.74M 37.95M 42.17M 50.18M
FLA496Hap2 - chr12:36042535-45804701 (9.76Mb)
36.04M 36.86M 37.68M 38.50M 39.32M 40.14M 40.96M 41.78M 42.61M 43.43M 44.25M 45.80M
PER226Hap1 - chr12
1 4.32M 8.64M 12.96M 17.28M 21.60M 25.92M 30.24M 34.57M 38.89M 43.21M 51.42M
PER226Hap1 - chr12:37986388-47748555 (9.76Mb)
37.99M 38.81M 39.63M 40.45M 42.09M 42.91M 43.73M 44.55M 45.37M 46.19M 47.75M
PER226Hap2 - chr12
1 4.22M 8..43M 12.65M 16.87M 21.09M 25.30M 29.52M 33.74M 37.95M 42.17M 50.18M
PER226Hap2 - chr12:36042535-45804701 (9.76Mb)
36.04M 36.86M 37.68M 38.50M 39.32M 40.14M 40.96M 41.78M 42.61M 43.43M 44.25M 45.80M
TreeCassavaHap1 - chr12
1 4.32M 8.64M 12.96M 17.28M 21.60M 25.92M 30.24M 34.57M 38.89M 43.21M 51.42M
TreeCassavaHap1 - chr12:37986388-47748555 (9.76Mb)
37.99M 38.81M 39.63M 40.45M 42.09M 42.91M 43.73M 44.55M 45.37M 46.19M 47.75M
TreeCasssavaHap2 - chr12
1 4.22M 8..43M 12.65M 16.87M 21.09M 25.30M 29.52M 33.74M 37.95M 42.17M 50.18M
TreeCasssavaHap2 - chr12:36042535-45804701 (9.76Mb)
36.04M 36.86M 37.68M 38.50M 39.32M 40.14M 40.96M 41.78M 42.61M 43.43M 44.25M 45.80M

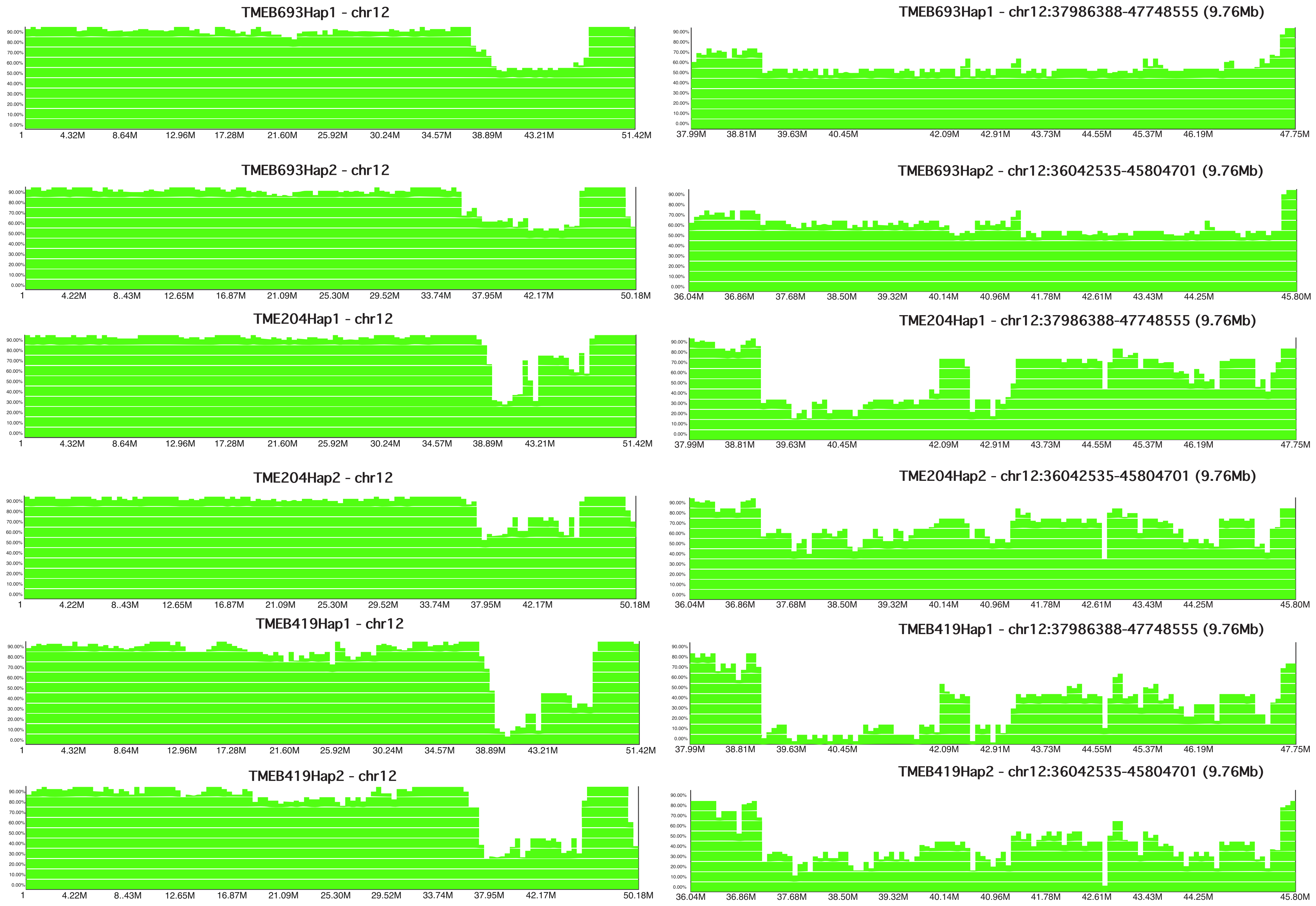
TMEB693Hap1 - chr12
1 4.32M 8.64M 12.96M 17.28M 21.60M 25.92M 30.24M 34.57M 38.89M 43.21M 51.42M
TMEB693Hap1 - chr12:37986388-47748555 (9.76Mb)
37.99M 38.81M 39.63M 40.45M 42.09M 42.91M 43.73M 44.55M 45.37M 46.19M 47.75M
TMEB693Hap2 - chr12
1 4.22M 8..43M 12.65M 16.87M 21.09M 25.30M 29.52M 33.74M 37.95M 42.17M 50.18M
TMEB693Hap2 - chr12:36042535-45804701 (9.76Mb)
36.04M 36.86M 37.68M 38.50M 39.32M 40.14M 40.96M 41.78M 42.61M 43.43M 44.25M 45.80M
TME204Hap1 - chr12
1 4.32M 8.64M 12.96M 17.28M 21.60M 25.92M 30.24M 34.57M 38.89M 43.21M 51.42M
TME204Hap1 - chr12:37986388-47748555 (9.76Mb)
37.99M 38.81M 39.63M 40.45M 42.09M 42.91M 43.73M 44.55M 45.37M 46.19M 47.75M
TME204Hap2 - chr12
1 4.22M 8..43M 12.65M 16.87M 21.09M 25.30M 29.52M 33.74M 37.95M 42.17M 50.18M
TME204Hap2 - chr12:36042535-45804701 (9.76Mb)
36.04M 36.86M 37.68M 38.50M 39.32M 40.14M 40.96M 41.78M 42.61M 43.43M 44.25M 45.80M
TMEB419Hap1 - chr12
1 4.32M 8.64M 12.96M 17.28M 21.60M 25.92M 30.24M 34.57M 38.89M 43.21M 51.42M
TMEB419Hap1 - chr12:37986388-47748555 (9.76Mb)
37.99M 38.81M 39.63M 40.45M 42.09M 42.91M 43.73M 44.55M 45.37M 46.19M 47.75M
TMEB419Hap2 - chr12
1 4.22M 8..43M 12.65M 16.87M 21.09M 25.30M 29.52M 33.74M 37.95M 42.17M 50.18M
TMEB419Hap2 - chr12:36042535-45804701 (9.76Mb)
36.04M 36.86M 37.68M 38.50M 39.32M 40.14M 40.96M 41.78M 42.61M 43.43M 44.25M 45.80M

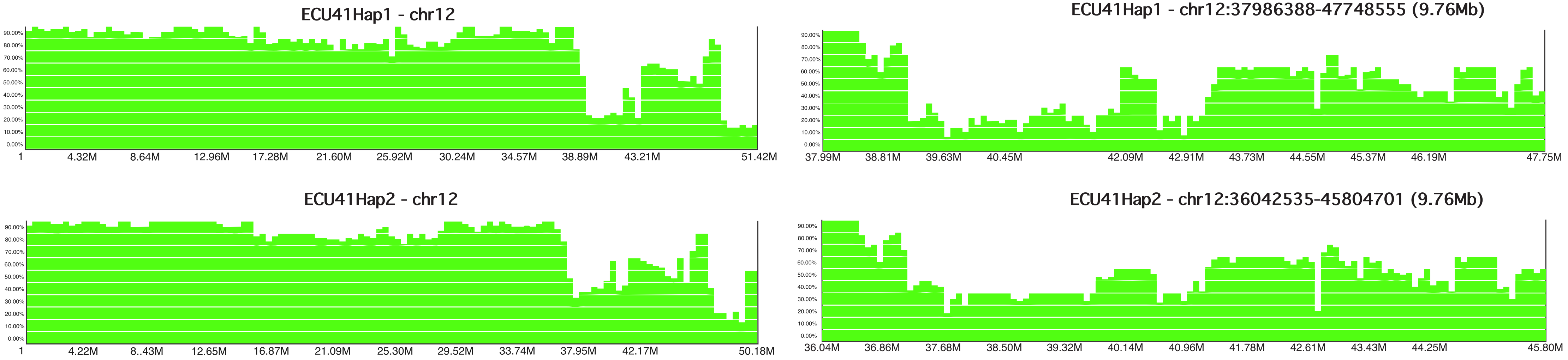

Fig. S2: Histogram of reads coverage plots for both haplotypes across the 16 cassava cultivars on chromosome 12 and insertion region

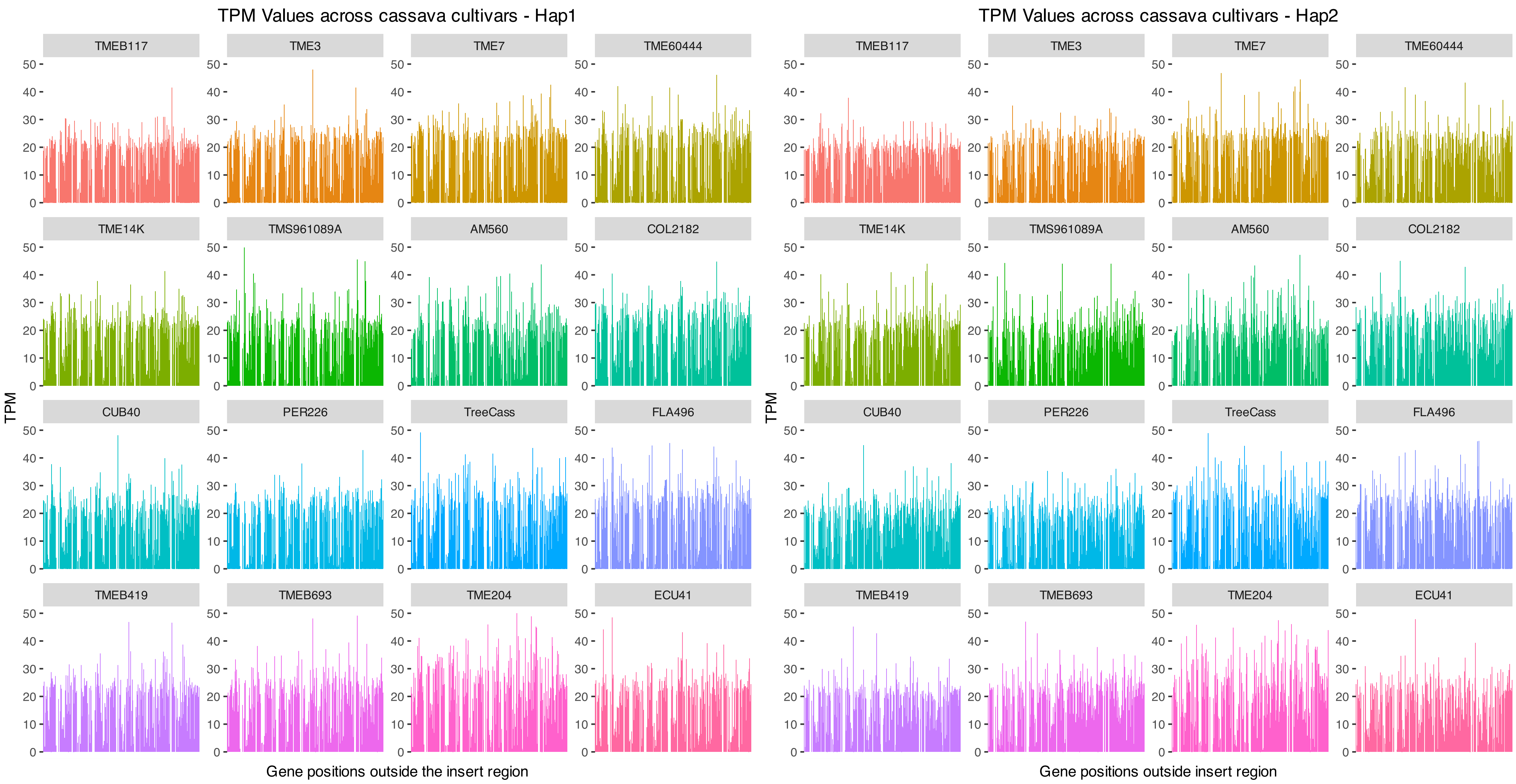

Fig. S3: Gene-by-gene bar plots of gene features outside the insert region 8Mb - (chr12:1,000,000-18,000,000) on both haplotypes

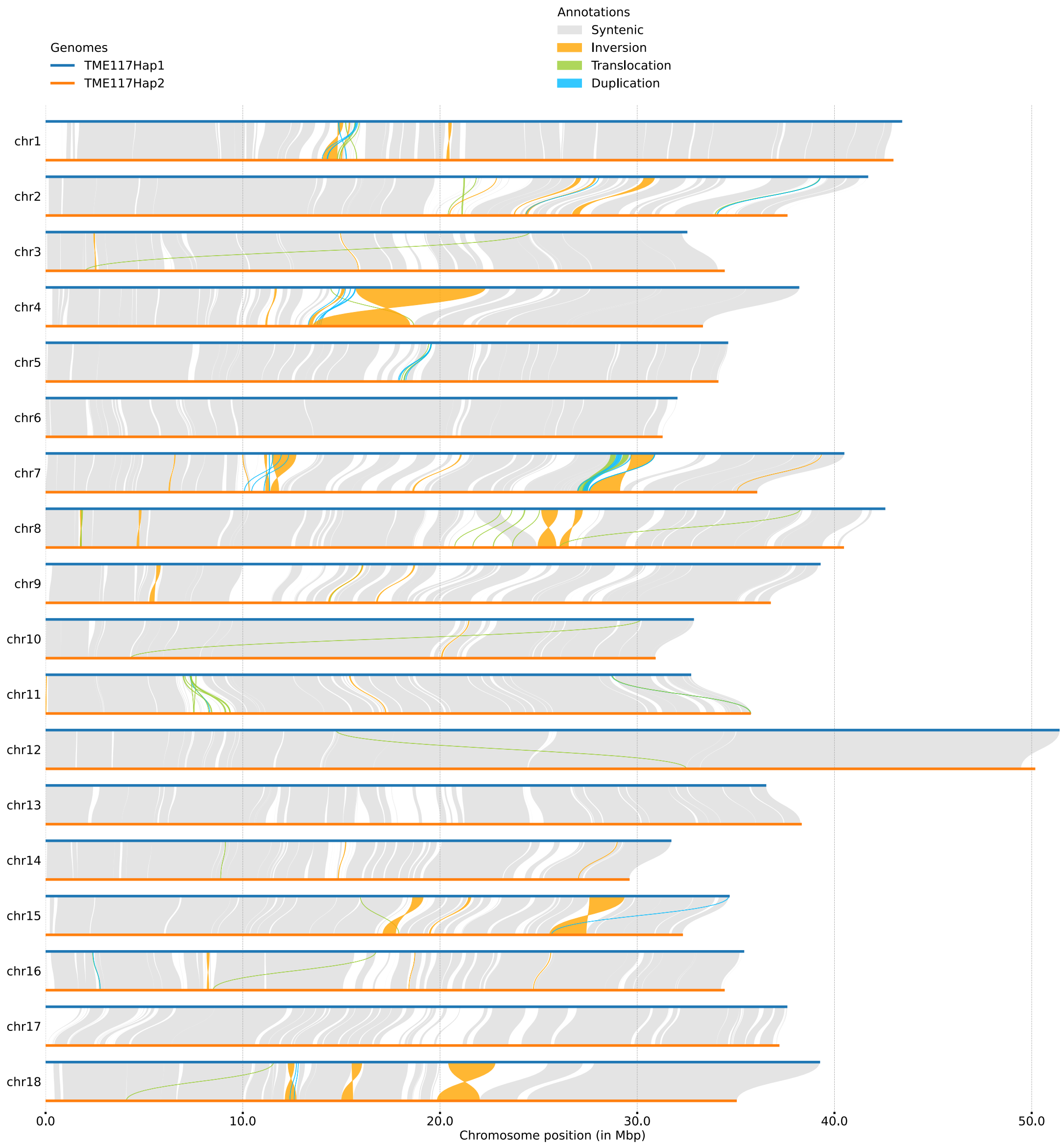

Fig. S4: Haplotypic structural variation within the TMEB117 genome

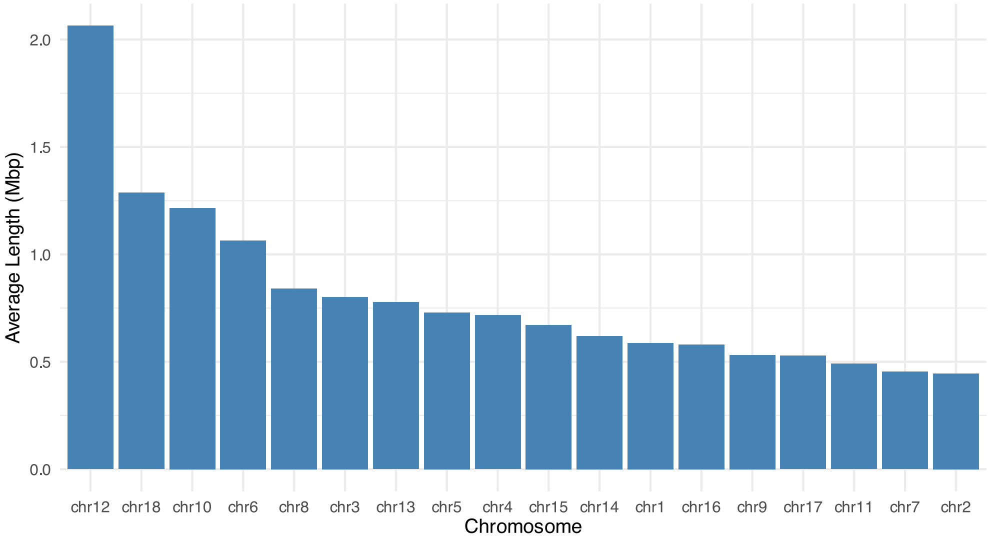

Fig. S5: Average length of synteny blocks per chromosome

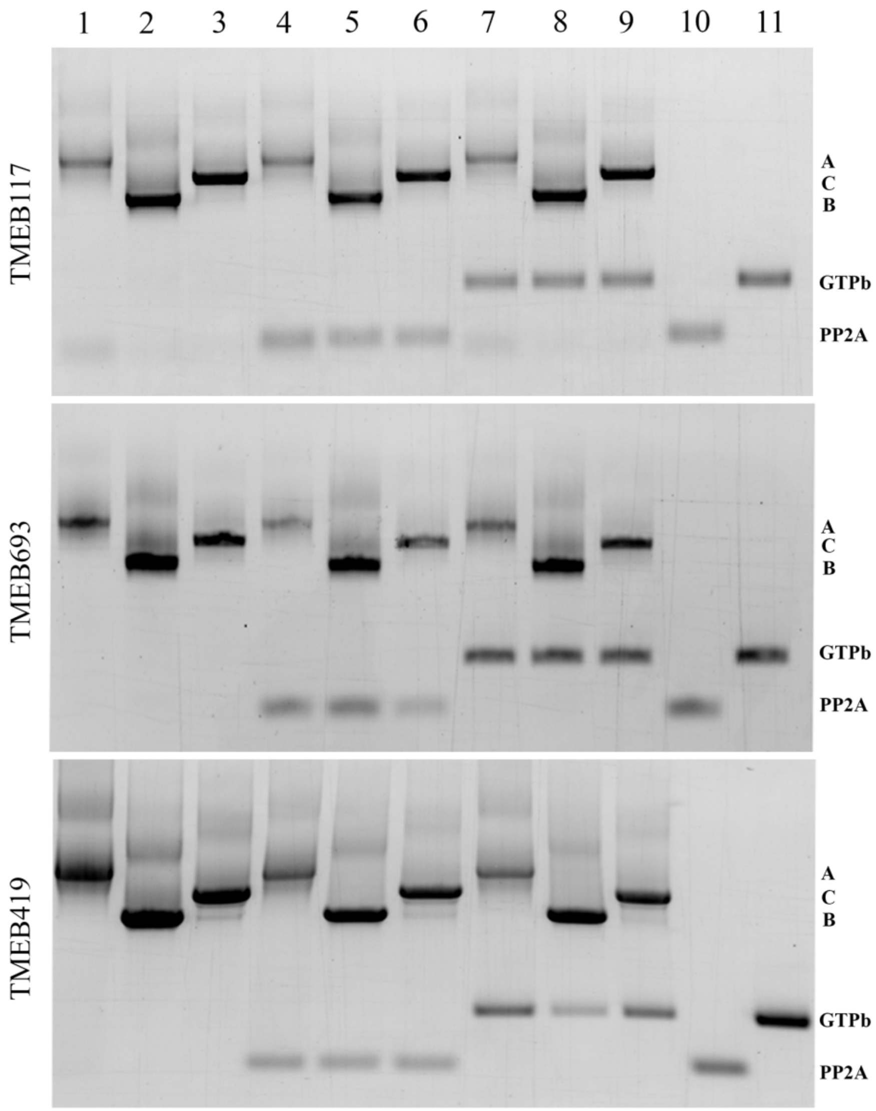

Lane 1: fragment A obtained with primers A (F and R)
Lane 2: fragment B obtained with primers B (F and R)
Lane 3: fragment C obtained with primers C (F and R)
Lane 4: fragment A and PP2A obtained with primers A (F and R) and primers PP2A (F and R)
Lane 5: fragment B and PP2A obtained with primers B (F and R) and primers PP2A (F and R)
Lane 6: fragment C and PP2A obtained with primers C (F and R) and primers PP2A (F and R)
Lane 7: fragment A and GTPb obtained with primers A (F and R) and primers GTPb (F and R)
Lane 8: fragment B and GTPb obtained with primers B (F and R) and primers GTPb (F and R)
Lane 9: fragment C and GTPb obtained with primers C (F and R) and primers GTPb (F and R)
Lane 10: PP2A obtained with primers PP2A (F and R)
Lane 11: GTPb obtained with primers GTPb (F and R)

Fig. S6: Electrophoresis gel analysis of PCR products. Insertion region fragments amplification from cassava genotypes TMEB117, TMEB693, TMEB419. Amplified genotypes are marked on the left side of the gel image panel. A list of primers used in this experiment is in Table S3.

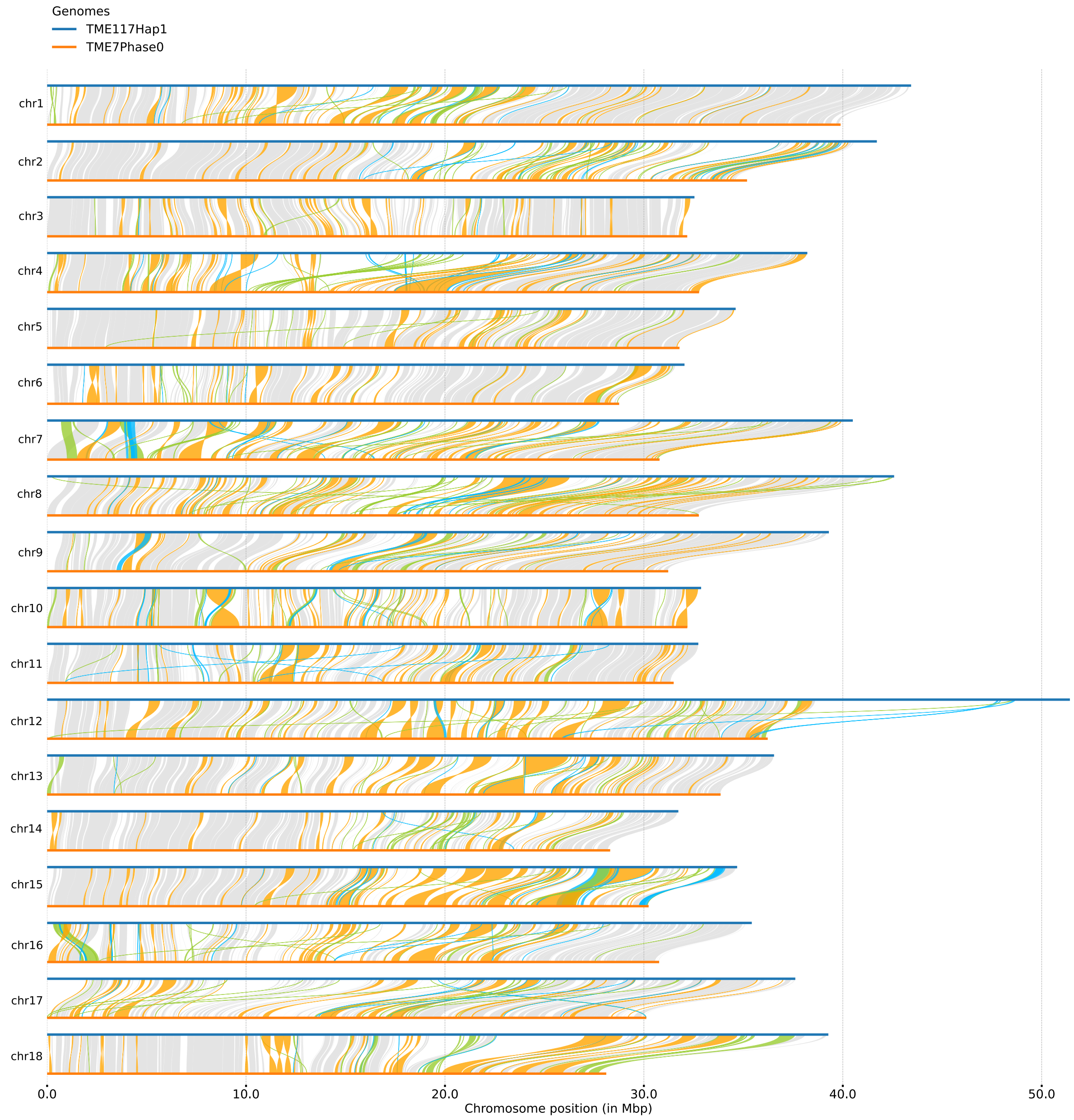

Fig. S7: Synteny plots compared between TMEB117 Hap1 with TME7Phase0

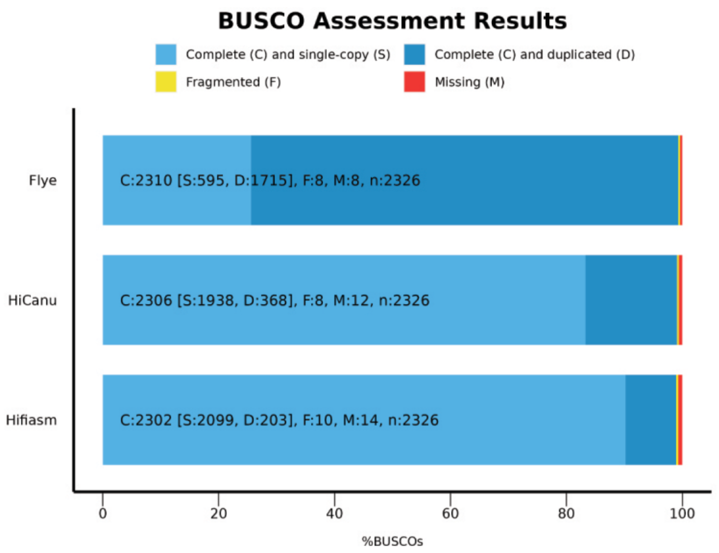

Fig S8: BUSCO results for Flye, HiCanu and hifiasm assemblies
